# Global Profiling of Genes Expressed in the Silk Glands of Philippine-Reared Mulberry Silkworms *(Bombyx mori)*

**DOI:** 10.3390/insects13080669

**Published:** 2022-07-24

**Authors:** Pauline Nicole O. de la Peña, Adria Gabrielle D. Lao, Ma. Anita M. Bautista

**Affiliations:** National Institute of Molecular Biology and Biotechnology, University of the Philippines Diliman, Quezon City 1101, Philippines; podelapena1@up.edu.ph (P.N.O.d.l.P.); adlao@up.edu.ph (A.G.D.L.)

**Keywords:** silkworm, *Bombyx mori*, RNA-seq, silk gland, transcriptome analysis

## Abstract

**Simple Summary:**

The sericulture industry in the Philippines remains to be enhanced through efforts to improve local strains of the silkworm *Bombyx mori,* such as selective hybridization. Genetic trait markers could help improve breeding; however, there is a scarcity of genetic data on local strains. This study aims to bridge this gap by analyzing the gene expression profiles of Philippine-reared silkworm strains through next-generation sequencing. Transcriptome assemblies were generated, and gene expressions were compared between silkworm strains reared in different temperatures, revealing genes that may be important for development in a tropical environment. This study is the first to provide transcriptome datasets for the Philippine-reared *B. mori* strains, which may serve as a resource for improving local strains and increasing silk production.

**Abstract:**

RNA sequencing was used to assemble transcriptome data for Philippine-reared silkworm and compare gene expression profiles of strains reared in high- and low-temperature environments. RNA was isolated from the silk glands of fifth instar larvae and mRNA-enriched libraries were sequenced using Illumina NextSeq 500. Transcriptome reads were assembled using reference-based and de novo assemblers, and assemblies were evaluated using different metrics for transcriptome quality, including the read mapping rate, E90N50, RSEM-eval, and the presence of single-copy orthologs. All transcriptome assemblies were able to reconstruct >40,000 transcripts. Differential expression analysis found 476 differentially expressed genes (DEGs; 222 upregulated, 254 downregulated) in strains reared in different temperatures. Among the top DEGs were myrosinase, heat shock proteins, serine protease inhibitors, dehydrogenases, and regulators of the juvenile hormone. Validation of some of the top DEGs using qPCR supported the findings of the in silico analysis. GO term enrichment analysis reveals an overrepresentation of GO terms related to nucleotide metabolism and biosynthesis, lipid and carbohydrate metabolic processes, regulation of transcription, nucleotide binding, protein binding, metal binding, catalytic activity, oxidoreductase activity, and hydrolase activity. The data provided here will serve as a resource for improving local strains and increasing silk production of Philippine-reared *B. mori* strains.

## 1. Introduction

The mulberry silkworm *Bombyx mori* is an economically important insect for the commercial production of silk, which is highly valued for its texture and luster, and for being one of the strongest natural fibers [1]. Silk has many uses such as in garments, upholstery, parachute linings, insulation, and as suturing material in surgeries. It is also considered a promising biomaterial in tissue engineering due to its mechanical strength and biocompatibility [2].

Since sericulture was introduced to the Philippines in the 1970s, local silkworm stocks have originated from Chinese, Japanese, and European strains [3]. Previous studies determined that temperate strains produce finer and stronger silk fibers, while tropical strains are more robust and disease-resistant but produce coarser and weaker silk fibers [4]. There have been efforts to breed hybrids that are acclimatized to the Philippine climate with desired phenotypic traits such as a high hatch rate, cocoon yield, cocoon weight, and silk filament quality [5,6,7]. Local silk production, however, has been reported to continue lagging behind demand, with 10 metric tons of demand annually and only 1 metric ton of raw silk production [8]. Low production has been attributed to the quality of local silkworm strains and poor rearing management [9]. The low silk yield may be improved by understanding how it is produced. The molecular mechanisms behind the different silk yields in silkworm strains, for example, need to be investigated as they are not yet well-understood [10,11]. Knowledge to be generated from molecular studies may therefore help in breeding local silkworm strains with a capacity to produce high silk yield. Markers associated with high silk yield and other economically important traits may also be developed for marker-assisted selection and breeding. Thus, this endeavor would need genetic resources, which can be sourced from genome and transcriptome sequences of foreign strains of *B. mori* in public databases such as the Silkworm Genomic Database (SilkDB) [12], KAIKObase [13], SilkTransDB (transcriptome) [14], SilkSatDb (microsatellites) [15], and BmTEdb (transposable elements) [16].

While there are already several genomic resources for the silkworm, transcriptome analysis can be used to gain insight into the functional elements of the genome and its biological pathways. One such technique is RNA sequencing (RNA-seq), which utilizes next-generation sequencing to provide high-throughput and high-resolution data to analyze expressed genes in a cost-efficient manner. RNA-seq can be used to identify novel transcripts, splice variants, and single nucleotide polymorphisms [17]. It also allows quantification over a dynamic range since it detects both highly and lowly expressed genes, making it ideal for gene expression analysis [18]. Recently, RNA-seq has been used by Zhang et al. to reveal gene expression changes in silkworms exposed to hydrogen sulfide [19]. Yokoi et al. also used RNA-seq to generate reference transcriptome data for major tissues of the silkworm p50T strain [20]. However, available transcriptome resources currently lack representative sequences from Philippine-reared *B. mori* strains; hence, the need to generate such resources is deemed important.

This study aimed to generate transcriptome resources for Philippine-reared mulberry silkworms, which can be utilized in future studies for its potential industrial valorization and to address the issue of low silk yield in the Philippines. This investigation focused on genes found in the silk glands of different strains of Philippine-reared silkworm.

## 2. Materials and Methods

### 2.1. Insect and Tissue Collection

Silkworms reared at the Philippine Textile Research Institute Technology Center in Misamis Oriental (PTRI-TCMO, Villanueva, Misamis Oriental) and the Technology Center of the Department of Science and Technology Cordillera Administrative Region (DOST-CAR, Benguet) were collected in 2018. The ambient temperature in TCMO and CAR at the time of collection was 31.7 °C and 23.9 °C, respectively. For each site, priority strains (MO204, LAT51, ITA) and a poorly performing strain (DMMMSU119) were used as representative strains. The strains were classified by the rearing facilities as either priority strains or poorly performing strains based on their rearing performance (hatching ratio, larval mortality, and pupation ratio). Economic characteristics such as silk yield and quality were not considered in the classification of priority strains. Three individuals per strain were used as bioreplicates. Silkworms were collected on the 3rd day of the 5th instar larval stage and were characterized morphologically. Larvae were preserved by flash-freezing in liquid nitrogen and stored at −80 °C until ready for dissection. Larvae were dissected in a petri dish on ice to collect whole silk glands. Silk glands were chopped using sterile surgical blades and homogenized mechanically using a mortar and pestle.

### 2.2. RNA Extraction and Sequencing

Total RNA was extracted from 100 µg of the tissue with the TRIzol reagent (Invitrogen, Life Technologies, Carlsbad, CA, USA). Total RNA was treated with TURBO DNAse (Ambion, Life Technologies, Carlsbad, CA, USA) and purified with the RNA Clean and Concentrator kit (Zymo Research, Irvine, CA, USA). The quality of RNA extracts was visually estimated by running them in 1% agarose gel at 90 V for 40 min. RNA purity was estimated by measuring the 260/280 and 260/230 absorbance ratios in a NanoDrop 2000/2000c Spectrophotometer (Thermo Fisher Scientific, Wilmington, DE, USA), and RNA was quantified by fluorometry using a Qubit RNA BR Assay kit (Thermo Fisher Scientific) according to the manufacturer’s instructions. Finally, extracts were analyzed in the Agilent 2200 TapeStation (Agilent Technologies, Santa Clara, CA, USA) to evaluate the RNA Integrity Number (RIN). Samples with RIN ≥ 6 were used for library preparation and sequencing.

The RNA samples were diluted and used as input (0.1–4 µg) for mRNA-enrichment library preparation using the TruSeq Stranded RNA Library Prep Kit (Illumina, San Diego, CA, USA). Twelve libraries were prepared (4 strains × 3 bioreplicates) with fragment sizes ranging 131–279 bp. Libraries were denatured, diluted to 1.8 pM, and combined with a spike-in of 1.0% PhiX sequencing control. The libraries were sequenced in the Philippine Genome Center using a NextSeq 500 (Illumina, San Diego, CA, USA) platform and a NextSeq v2 high output kit (Illumina, San Diego, CA, USA) with 300 cycles (2 × 150 paired-end sequencing).

### 2.3. Preprocessing, Alignment, and Transcriptome Assembly

Illumina bcl2fastq (v. 2.2.0) was used to convert sequencing base call files to FASTQ format. FastQC was used to evaluate reads and visualize read quality. Raw reads were processed using Trimmomatic (0.39) and fastp (0.20.0) to trim adapter sequences and filter low-quality reads. 

The splice-aware aligner STAR (2.7.0f) was used to align reads to the *B. mori* reference genome from NCBI (RefSeq GCF_000151625.1). The BAM file was processed with RNA-SeQC (1.1.9) to evaluate RNA-Seq data bias based on the total read count, coverage, and expression correlation (RPKM-based estimation of expression levels). Another aligner, HISAT (2.1.0), was used for alignment in preparation for generating the StringTie assembly. HISAT alignment files were used for transcriptome assembly evaluation and expression quantification.

Reference-based assembly was performed using Cufflinks (2.2.1) and StringTie (1.3.6). In addition to reference-based assembly, de novo assembly was also performed using Trinity (2.8.6). The percentage of aligned reads was determined using Bowtie. Contig statistics (number of transcripts, N50, mean, and median contig length) were generated using Trinitystats.pl. Assemblies were evaluated using DETONATE (1.11) to estimate their RSEM-EVAL scores. The completeness of the assemblies was evaluated using BUSCO (v. 3.0.2).

### 2.4. Expression Quantification and Differential Expression Analysis

FeatureCounts (1.4.6-p5) was used for gene-level quantification and DESeq2 (1.30.1) was used for gene-level differential expression analysis. Differentially expressed genes (DEGs) were filtered based on the false discovery rate (FDR)/adjusted *p*-value < 0.1 and |log_2_ fold change| > 1. Figures to visualize DEGs were generated from the plotMA function of DESeq2 and R packages EnhancedVolcano (1.8.0) and pheatmap (1.0.12). 

The annotation of DEGs was performed against the NCBI nr protein database with an e-value cut-off of 1 × 10^−5^. GO terms were mapped to DEGs by matching the protein IDs to the GO annotations in the newest release of the *B. mori* genome annotations from KAIKObase 4.1.0 (https://kaikobase.dna.affrc.go.jp/KAIKObase_download.html (accessed on 4 April 2021). The R package goseq (1.42.0) was used to determine GO term enrichment among DEGs. Significantly enriched GO terms were summarized and visualized using REVIGO (http://revigo.irb.hr/ (accessed on 8 April 2021).

### 2.5. Quantitative Real-Time PCR (qPCR)

Quantitative real-time PCR assays were carried out using the Bio-Rad CFX96™ Touch system (Bio-Rad Laboratories, Inc., Hercules, CA, USA). Each 10-μL reaction contained 5 μL of 2X iTaq Universal SYBR Green Supermix (Bio-Rad Laboratories, Inc., Hercules, CA, USA), 5 pmol each of forward and reverse primers, and 25 ng of cDNA, following a thermal profile comprising initial denaturation at 95 °C for 5 min, 40 cycles of denaturation at 95 °C for 10 s, annealing and extension at 60 °C for 30 s, and a melt curve assay from 95 °C to 65 °C in 0.5 °C stepwise increments. Primers for select genes differentially expressed between CAR and TCMO were designed using Primer-BLAST and are shown in Table 1. 

Total RNA was extracted from silkworms reared at the PTRI-TCMO and PTRI-CAR, followed by TURBO DNase treatment as previously described. RNA from two individual silkworms from each location was pooled and used to generate cDNA using the ProtoScript II First Strand cDNA Synthesis Kit (New England Biolabs, Ipswich, MA, USA) according to the manufacturer’s recommendations. The cDNA was quantified by fluorometry using the Qubit ssDNA Assay kit (Thermo Fisher Scientific). Serial dilutions (10×) of pooled cDNA samples were prepared to generate a standard curve for each primer set used for the qPCR assays, starting at 100 ng of cDNA. The standard curves were used in conjunction with cycle threshold values to calculate starting quantities of the transcripts, and the expression levels and fold changes of each gene were normalized to *B. mori* ribosomal protein 49 [20]. All reactions were performed in triplicate. Calculations were performed using the Bio-Rad CFX Manager (v. 3.1). Statistical significance was determined using Student’s t-test with GraphPad Prism 6.

## 3. Results

### 3.1. Selection of Representative Silkworm Strains

Representative strains from the two collection sites were selected based on their phenotypic and economic characteristics (Table 2). Each strain can be differentiated by its larval markings. MO204 and DMMMSU119 are milky white, while LAT51 and ITA are beige.

The rearing sites had different conditions, particularly temperature and relative humidity (RH), the most important environmental factors in silkworm rearing. During sample collection, the temperature in TCMO was higher by 7.8 ± 0.1 °C and RH was also higher by 12%. 

### 3.2. RNA Sequencing and Quality Control

High-quality RNA was extracted (RIN ≥ 6), and the sequencing libraries had fragment sizes ranging from 131 to 279 bp, as determined by the Agilent TapeStation. Sequencing yielded 50.1 Gb of 169.2 M reads passing filter, with 96.1% of reads having >Q30.

The number of raw reads from RNA sequencing is shown in Table 3. All ITA bioreplicates yielded lower than the expected number of reads. Because the number of reads was deemed insufficient, ITA was no longer included in downstream analyses.

**Figure 1:**
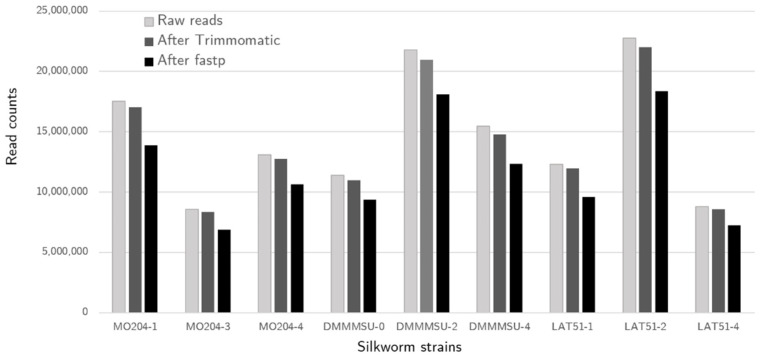
*Bombyx mori* raw read counts and proportion of reads after processing.

Trimmomatic was used for trimming Illumina adapters that were ligated to tag each sample during sequencing, and for quality trimming on leading and trailing sequences. Sequences that were under 36 bp were also dropped. A sliding window of 4:20 was used, where the sequences will be trimmed if the quality drops below Q20 in a 4-base window. After processing with Trimmomatic, most sequences were still retained, indicating that only a small number of sequences were dropped due to having low-quality bases (Figure 1). After Trimmomatic processing, poly G sequences still appeared in overrepresented sequences in FastQC, particularly in forward reads. Poly G sequences are artifacts in NextSeq sequencing due to its two-color filter that calls for G bases when there is no emission detected. Poly G sequences were filtered out using fastp, after which 77.9–83.1% of the raw reads were retained.

### 3.3. RNA-seq Read Alignment to the Reference Genome

**Figure 2:**
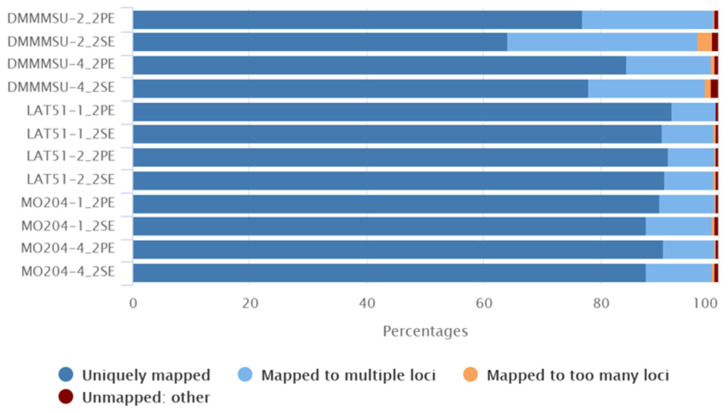
Summary of STAR alignment statistics of *Bombyx mori* RNA-seq reads to the reference genome. (PE: Paired-end reads, SE: Single-end reads). Generated using STAR.log files and MultiQC [23].

Two samples per strain with the most read counts were aligned to the *B. mori* reference genome RefSeq GCF_000151625.1 [21] using the splice-aware aligner STAR. Most single and paired reads were uniquely mapped to the reference with only a small percentage (0.11–0.53%) of unmapped reads (Figure 2). The DMMMSU replicates had a higher percentage of reads that were mapped to multiple loci (22.73% and 14.70% for DMMMSU-2 and DMMMSU-4, respectively), while for LAT51 and MO204 replicates, more than 90% of reads were uniquely mapped.

Moreover, most of the splice sites detected by STAR were in the GTF annotation of the reference genome. It has been reported that STAR generates a large number of putative novel splice sites [22], but in this case, the splice sites were validated by the reference genome annotation.

Samtools flagstat was also run to analyze data from the FLAG field of the generated BAM files. According to flagstat, most of the mapped reads were properly paired and mapped with their mate. No duplicates were detected, but secondary alignments were produced (ranging from 9.6 to 31%). This coincides with the STAR statistics indicating that there were several reads mapped to multiple loci (ranging from 7.43 to 22.73%). In the BAM files of the paired-end reads, less than 1% were singletons, and 0 reads had mates mapped to a different chromosome.

### 3.4. Transcriptome Assembly and Evaluation Metrics

**Figure 3:**
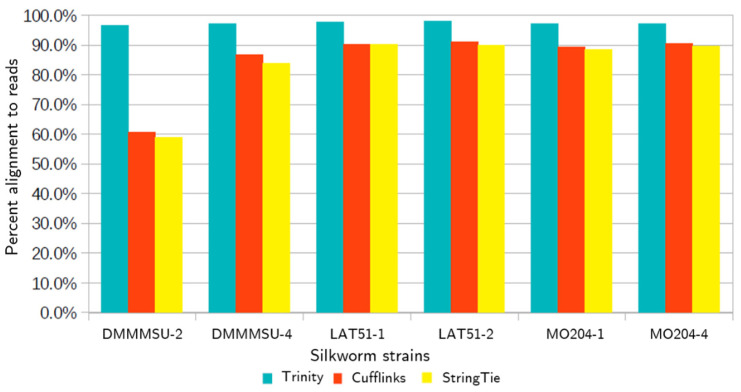
Overall alignment rate of RNA-seq reads to the transcriptome assemblies.

After assembling the reads using different assemblers, the assemblies were mapped back to the input reads to evaluate their read support. Figure 3 shows that the reads had the highest alignment rates with the Trinity assembly, while the Cufflinks and StringTie assemblies had comparable alignment rates. Since the reads from the DMMMSU samples had a lower percentage of uniquely mapped reads, it could be expected that the reference-based assemblies would have less support from these samples.

Transcriptome assemblies are also evaluated based on the number and length of unigenes (unique sequences reconstructed in the assembly; includes not only genes but all assembled contigs). According to the contig statistics in Table 4, the Trinity assembly had the lowest N50 (1313) and lowest median contig length (468). Cufflinks had the highest contiguity with the highest N50 (3414), highest median contig length (1738), and average contig length (2305.35). However, in a transcriptome assembly, long contig lengths and high N50 are not as important as in genome assemblies. The most highly expressed transcripts are not necessarily the longest ones, and the majority of transcripts are expected to have low expression levels. Transcript lengths are also not indicative of a good transcriptome assembly since transcripts may be present in a wide range of sizes.

Trinity assemblies had the highest overall alignment rates (96.60–98.00%), while the alignment rate for the Cufflinks and StringTie assemblies were comparable (60.5–91.10% and 58.80–90.2%, respectively).

A metric more appropriate for transcriptome assemblies is the ExN50 statistic, which represents N50 based on the most highly expressed transcripts that represent x% of the normalized expression data (e.g., E90N50 is the N50 value for 90% of the normalized expressed transcripts, excluding the lowly expressed transcripts) [24]. To obtain the ExN50 scores, the expression data were first normalized using kallisto, then count matrices were generated. Finally, the Trinity scripts contig_ExN50_statistic.pl and plot_ExN50_statistic.Rscript were run to compute and visualize ExN50 contig statistics.

**Figure 4:**
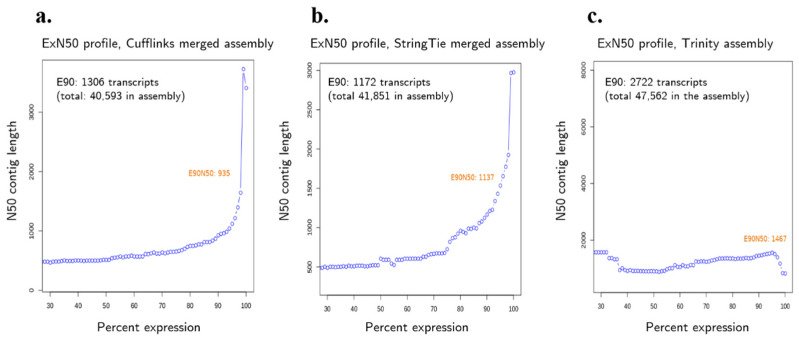
ExN50 profiles of the (**a**) Cufflinks, (**b**) StringTie, and (**c**) Trinity merged *Bombyx mori* transcriptome assemblies.

Comparing the N50 of the assemblies with their E90N50, a reversal is observed. When all contigs were considered (E100), Cufflinks and StringTie had a higher N50 than Trinity, indicating longer contig lengths. However, when N50 is confined to 90% of expression data, the Trinity assembly resulted in a higher value for E90N50. This suggests that lowly expressed genes make up longer contigs in reference-based assemblers but not in the de novo assembler. The ExN50 profiles in Figure 4 show that the peak N50 occurs at higher Ex values, indicating that the sequencing depth was enough to assemble the lowly expressed transcripts.

A reference-free evaluation metric for transcriptome assemblies was developed by Li et al. [25] in the package DETONATE (De novo Transcriptome RNA-seq Assembly with or without the Truth Evaluation). DETONATE has two component packages, RSEM-EVAL and REF-EVAL. RSEM-EVAL is based on the RSEM (RNA-Seq by Expectation Maximization) algorithm. RSEM-EVAL is based on a probabilistic model that combines different factors including the compactness of the assembly and read support [25]. The RSEM-EVAL score is a log joint probability based on three components: Likelihood of the assembly, assembly prior, and a Bayesian information criterion penalty. Penalties are imposed when assemblies have too many bases or contigs or have an unusual distribution of contig lengths relative to the expected read coverage. RSEM-EVAL scores are always reported to be negative, but higher scores are indicative of better assemblies. Out of the three transcriptome assemblies, Trinity obtained the highest RSEM-EVAL score (Table 5). Aside from the RSEM-EVAL scores, REF-EVAL scores are also reported in Table 5. REF-EVAL reports the recall and precision of the assemblies at the contig and nucleotide levels. The recall is the fraction of reference elements (contigs, scaffolds, nucleotides) that are recovered in the assembly, while precision is the fraction of assembly elements that recover a reference element. The F_1_ score represents the harmonic mean of the recall and precision scores:

(1) $$F_{1}=\frac{2\times recall\times precision}{recall+precision}.$$

The k-mer compression (*KC*) score is computed by the formula:

(2) KC=WKR (weighted kmer recall)−ICR (inverse compression rate),

where *WKR* measures an assembly’s recall of the k-mer content in the reference, with each k-mer weighted by relative frequency, and *ICR* measures the compression of the RNA-seq data.

**Figure 5:**
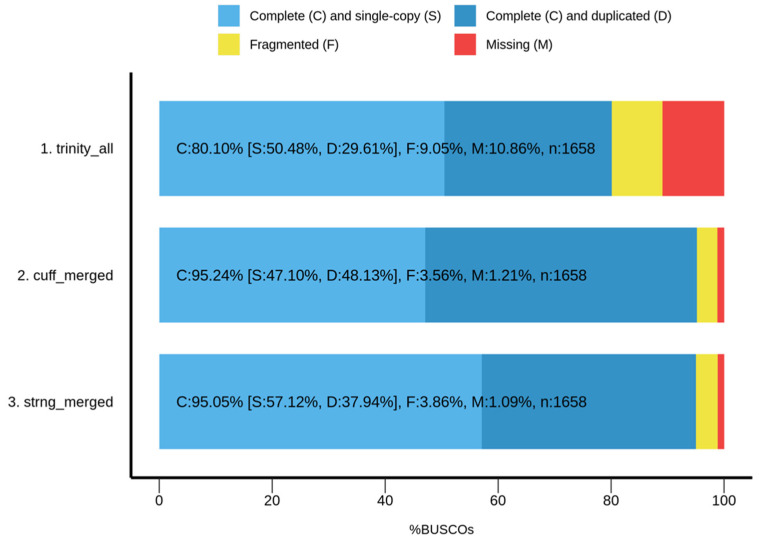
Measure of completeness of transcriptome assemblies from *Bombyx mori* strains using BUSCO (dataset: insecta_odb9).

To evaluate the completeness of assemblies, Benchmarking Universal Single-Copy Orthologs (BUSCO) was employed using the dataset insecta_odb9 (2016). BUSCOs are core genes from OrthoDB that are expected to be present as single copies in orthologous groups. Recovered genes are classified as ‘complete’ when their lengths are within two standard deviations of the group mean length; ‘fragmented’ when they are only partially recovered; and ‘missing’ if they are unrecovered [26]. Both reference-guided assemblies had a high percentage (>95%) of completely recovered BUSCOs, while the Trinity assembly had the lowest number of BUSCOs at 80.10% (Figure 5). The StringTie assembly had more single-copy and fewer duplicate orthologs. Duplicated BUSCOs should be rare and could indicate erroneous assembly of haplotypes [26]. Running BUSCO for unmerged transcriptome assemblies (per sample) resulted in a small percentage of duplicated BUSCOs, so the duplication may be due to heterozygous alleles that were not collapsed when the assemblies were merged.

To summarize the results of the different transcriptome assembly evaluation metrics, the Trinity de novo assembly performed best in terms of alignment rate, assembled the greatest number of transcripts with high contig length represented by E90N50, and had the best RSEM-eval score. However, when it comes to the completeness of assembly based on BUSCO, Cufflinks outperformed the other assemblers.

### 3.5. Expression Quantification, Normalization, and Differential Expression Analysis

The reads were counted according to gene-level features using FeatureCounts (1.4.6-p5), which is part of the R Subread package [27]. Quantification was run at the meta-feature level (gene level), where reads that overlap multiple exons of the same gene are counted exactly once, provided there is no overlap with another gene (i.e., exon spanning reads will be counted once). Read count normalization was performed with DE analysis using DESeq2’s size factor normalization. After running DESeq2, 1647 DEGs were identified within the threshold *p* < 0.1, but after FDR correction, only 476 DEGs were within the adjusted threshold *p_adj_* < 0.1. Factor levels were set by location, where TCMO is the reference condition and CAR is set as the contrast condition. Among the 476 DEGs, there were 222 upregulated genes and 254 downregulated genes in CAR strains.

**Figure 6:**
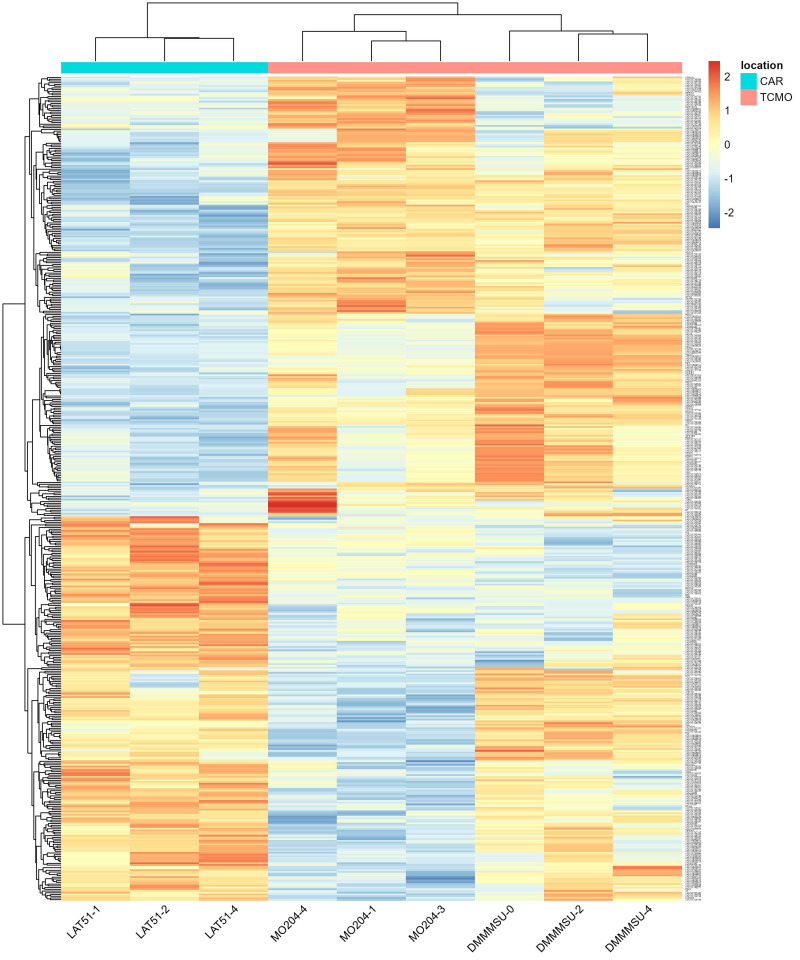
Heat map of the 112 DEGs from *Bombyx mori* with *p*_adj_ < 0.1. The values shown are scaled to the distances from the average of each row (gene).

A heat map of the 476 DEGs that passed the significance threshold is shown in Figure 6. Clustering of the DEGs by location is apparent in the heat map. The replicates for each strain show similar DEGs, except for a cluster of DEGs that are upregulated in bioreplicate MO204-4 but not in other MO204 replicates. This DEG cluster is composed of loci with uncharacterized proteins (LOC101741578, LOC101739373, LOC101736556, LOC101741319, LOC101738771, LOC101740400, LOC101735788). LOC101741578 has a conserved domain (COG5240 SEC21; vesicle coat complex COPI, gamma subunit) that is found in proteins involved in intracellular traffic and secretion [12]. Since these genes are highly upregulated in MO204-4 but not in other TCMO strains, this may be due to individual differences that were not taken into account (e.g., sex differences).

**Figure 7:**
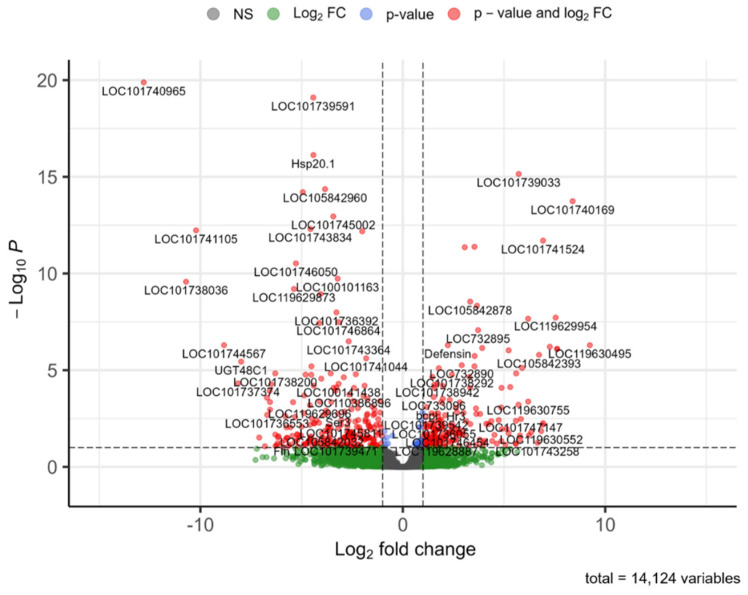
Volcano plot of DEGs from *Bombyx mori*, log fold change vs. −log *P*. Gray: Nonsignificant genes; green: Genes with significant fold changes but insignificant adjusted *p*-values; blue: Genes that passed the significance threshold according to adjusted *p*-value but did not have significant fold changes; red: Genes that have adjusted *p*-value < 0.1 and significant fold change (|log_2_ FC| > 1). Graph generated in R package EnhancedVolcano [28].

DEGs were filtered by the criteria *p*_adj_ < 0.1 and |log_2_ FC| > 1 to select statistically and biologically significant genes; genes passing this significance threshold are represented by red points in the volcano plot shown in Figure 7. The filtered DEGs with their corresponding gene and protein IDs are listed in Table S1.

### 3.6. Functional Annotation of Select DEGs

The top DEGs were selected based on statistical significance and high fold changes and are summarized in Table 6. This list includes fold changes, *p*_adj_ values, as well as the protein names (if available) and the functions derived from Pfam/InterPro annotations available in UniProt [29].

Gene Ontology (GO) terms were mapped to the gene sets by matching the protein IDs to the GO annotations in the newest release of *B. mori* genome annotations from KAIKObase (ver. 4.1.0, July 2020). Their annotation contains the newest predicted gene models, descriptions including InterPro IDs, GO terms, and best hit in the NCBI nr database. Out of 14,124 assayed genes, 11,792 were annotated with protein IDs from nr, and 3948 were annotated with GO terms.

GO term enrichment was determined using the R package goseq. Significantly overrepresented GO terms are summarized in Tables S1 and S3 for upregulated and downregulated genes, respectively. Among the upregulated genes, the biological processes that were enriched are related to nucleotide metabolism and biosynthesis, lipid and carbohydrate metabolic processes, and regulation of transcription. Among down-regulated genes, similar biological processes were found to be enriched, but the ontology for carbohydrate metabolic process stands out with a low *p*-value. For cellular components, there is no overlap of GO terms for up- and downregulated genes. For molecular functions, nucleotide, protein, and metal binding are enriched in upregulated genes, while DNA binding, ATP binding, and zinc binding are enriched in downregulated genes. Catalytic activity, oxidoreductase, and hydrolase activities are also enriched molecular functions found in both sets.

### 3.7. qPCR Validation of Select DEGs

**Figure 8:**
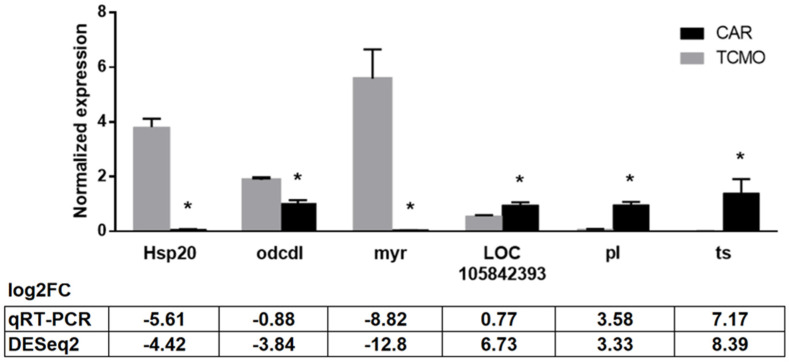
Results of qPCR validation of differentially expressed genes. Heat shock protein 20 (Hsp20), oxygen-dependent choline dehydrogenase-like (odcdl), and myrosinase 1 (myr) were downregulated in CAR vs. TCMO, while uncharacterized protein (LOC105842393), peroxidase-like (pl), and uncharacterized protein tetraspanin family (ts) were upregulated in CAR vs. TCMO. Log_2_FC values calculated from qPCR results are compared with values obtained during DESeq2 analysis. Error bars indicate standard deviation; asterisks denote significant change in CAR vs. TCMO (Student’s *t*-test), *p* < 0.05.

Among the selected top DEGs in Table 6, six were selected for validation using qPCR: Heat shock protein 20 (Hsp20), oxygen-dependent choline dehydrogenase-like (odcdl), myrosinase 1 (myr), peroxidase-like (pl), uncharacterized protein (LOC105842393), and uncharacterized protein tetraspanin family (ts). The first three were observed in silico to be downregulated in CAR vs. TCMO silkworms, while the last three were calculated to be upregulated. The results for the qPCR found Hsp20 to be significantly downregulated in CAR vs. TCMO samples (*p* < 0.0001), as were odcdl (*p* = 0.0005) and myr (*p* = 0.0008). LOC105842393 was found to be significantly upregulated in CAR vs. TCMO (*p* = 0.028), as were pl (*p* = 0.0001) and ts (*p* = 0.0093). These data support the results of in silico analysis (Figure 8). Additionally, log_2_FC values were calculated and compared with the values produced using DESeq2, as presented in Figure 8. While differences were found between the log_2_FC values between qPCR and DESeq2, these may be due to the individual gene expression differences between samples used for sequencing and samples used for qPCR. Despite these differences, the trends of downregulation or upregulation in CAR vs. TCMO samples observed during qPCR appear to support those found during in silico analysis.

## 4. Discussion

In this study, we compared gene expression in silk glands of *B. mori* strains reared in two different local facilities with different rearing temperatures. According to previous studies, the optimal temperature for silkworm growth is 20–28 °C and 23–28 °C for silk productivity [30]. The ambient temperature in the rearing sites during collection was 31.7 °C and 23.9 °C for TCMO and CAR, respectively. These data are based on bivoltine strains, although polyvoltine strains acclimatized in tropical countries are known to tolerate higher temperatures. Nevertheless, it has been shown that lower temperatures are better for silkworm productivity and larval duration [31].

Closely related to temperature is humidity, thus, an important factor to consider. It influences the withering of leaves in rearing beds, which affects larval feeding. However, there is no limiting range for humidity, and most insects can develop as long as they can control their water balance [30].

Among the downregulated genes in CAR were myrosinase, heat shock proteins, and serine protease inhibitors, which are related to defense and ensuring proper protein folding. These were downregulated in CAR strains likely because chaperone and protease inhibitors are not needed as much in lower temperatures. Aside from Hsp20.1, other heat shock proteins that are significantly downregulated are Hsp 68, Hsp 70, Hsp 23.7 precursor, and Hsp 25.4 precursor. 

On the other hand, there were upregulated genes found in CAR related to defense such as peroxidase-like protein, defensin, and other proteins related to oxidoreductase activity. Many of these protein products are yet to be characterized, but sequence motifs provide some hints about their function based on information from protein families and conserved domains.

There were also several dehydrogenases found among the DEGs, such as oxygen-dependent choline dehydrogenase-like protein, L-sorbose 1-dehydrogenase, glucose dehydrogenase [FAD, quinone], 15-hydroxyprostaglandin dehydrogenase, farnesol dehydrogenase, dihydropyrimidine dehydrogenase [NADP(+)], mitochondrial aldehyde dehydrogenase, and alcohol dehydrogenases. Dehydrogenases are a type of oxidoreductases that oxidize substrates by reducing an electron acceptor and are important for their role in detoxification of metabolites. Malate dehydrogenase (BmMDH1) and 6-phosphogluconate dehydrogenase (Bm6PGD) were previously found to be differentially expressed in Dazao silkworms exposed to different temperatures, which implies enhancement in energy metabolism and ATPase expression at low temperatures [32].

Another DEG of note is SOSS complex subunit B homolog (log_2_FC: −2.0042; *p*_adj_: −2.004 × 10^−13^). The SOSS complex binds to single-stranded DNA and is involved in DNA damage response, cell cycle checkpoint activation, homologous repair of double-stranded breaks, and ATM-dependent signaling pathways [33]. Downregulation of SOSS in CAR strains may indicate that there is less DNA damage response in silkworms reared in low-temperature environments.

Microvitellogenin (log_2_FC: 6.2283; *p*_adj_: 0.023) was one of the genes found upregulated in CAR strains, which encodes a low molecular weight (30 kDa) lipoprotein (30 K proteins, or 30 KPs). These lipoproteins are synthesized in the *B. mori* fat body and secreted in the hemolymph during the last instar larva stage. They are involved in several physiological processes, such as energy storage, embryonic development, and immune response [34]. Upregulation of microvitellogenin may indicate higher energy storage and enhanced immunoprotection in low-temperature strains [35,36].

Another upregulated DEG was the protein eyes shut (log_2_FC: 5.7860; *p*_adj_: 0.024), also known as EGF-like protein 10, an essential protein for the formation of photoreceptor cells in *Drosophila* [33]. Most EGF-like domains are found in extracellular domains of membrane-bound proteins or secreted proteins. Aside from its EGF-like domain, *B. mori* protein eyes shut also has a laminin G domain, which is associated with different functions such as cell adhesion, signaling, migration, assembly, and differentiation [37]. 

Three regulators of the juvenile hormone (JH) were found among the DEGs in this analysis: Juvenile hormone epoxide hydrolase-like protein 2 (Jheh-lp2), juvenile hormone epoxide hydrolase precursor (Jheh2), and cytosolic juvenile hormone binding protein 36 kDa subunit (Cjhbp). JHEH inactivates juvenile hormones by hydrolyzing the epoxide groups in JH. Differential expression of JH regulators could explain the observed differences in larval development between CAR and TCMO strains, wherein CAR strains have a longer larval duration (23 days for TCMO, 27 days for CAR). JH has long been known to affect silk production, and the application of JH analogs has been used to prolong the 5th instar stage to increase larval weight and silk secretion [38]. More recently, the JH regulatory pathway has been demonstrated to influence Fib-H expression through the action of transcription factors *Bmdimm* and *Bmsage* [39]. Zhou et al. [40] showed that JH biosynthesis and sexual maturation are delayed in cotton ballworm (*Helicoverpa armigera*) reared at 19 °C compared to those reared in higher temperatures. Liu et al. [41] showed that JH titers in Formosan termite (*Coptotermes formonasus*) were positively correlated with temperature. In contrast, Geister et al. [42] found no correlation between temperature and JH titers in the tropical butterfly *Bicyclus anynana*.

Caytaxin was downregulated in CAR strains (log_2_FC: −2.8609; *p*_adj_: 0.032). Caytaxin is a member of the BNIP2 (Bcl2-/adenovirus E1B nineteen kDa-interacting protein 2) protein family (pfam12496). Caytaxins interact with pro- and anti-apoptotic molecules in the cell and are involved in Rho GTPase regulation [43].

Another DEG found was dymeclin (log_2_FC: 1.7453; *p*_adj_: 0.030), which belongs to the Dymeclin (Dyggve-Melchior-Clausen syndrome protein) protein family (pfam09742) in plants and animals. Dymeclin proteins have a length of approximately 700 residues and contain many leucine and isoleucine in their conserved domain [12]. Mutations in human dymeclin cause Dyggve–Melchior–Clausen syndrome (DMC, MIM 223800), an autosomal recessive disorder. Dymeclin is a peripheral membrane protein dynamically associated with the Golgi apparatus. In *Caenorhabditis elegans*, a member of the Dymeclin protein family is hid1 (high-temperature-induced dauer-formation protein 1)*,* which encodes a highly conserved transmembrane protein that could be involved in vesicle secretion or intercellular signaling [44].

Previous comparative studies found similar enrichment in GO terms as those in this study. In one study comparing wild and domestic silkworms, oxidoreductase activity (GO: 0016491) was the only enriched GO term in four pairwise comparisons [11]. In another study comparing Chinese silkworm strains JingSong and Lan10, which have different rates of silk production, the dominant GO term was membrane-enclosed lumen under the cellular component [10].

## 5. Conclusions

The present study provides, for the first time, valuable information on the transcriptome of *B. mori* strains found in the Philippines. The differential expression analysis performed on silk glands of *B. mori* strains grown in different sites gives us insight into the processes and functions linked to the down- and up-regulated genes. This molecular information could be used as a source of trait markers for the improvement of local silkworm strains to enhance the sericulture industry in the Philippines. For future directions, aside from protein annotations and GO terms, KEGG pathway analysis may also be performed to further elucidate the functions of DEGs in silkworms and in silk production. It is also recommended to perform further quantitative PCR assays to validate the other bioinformatically obtained DEGs, in addition to the DEGs validated here. Furthermore, this study could be extended to transcriptome analysis of other *B. mori* strains, tissues, and developmental stages.

## Figures and Tables

**Table 1:** Primers used for qPCR validation of differentially expressed genes in *Bombyx mori* strains.

Primers	Sequences (5′ to 3′) Forward Reverse	Product Length (bp)	Protein ID
Downregulated genes (CAR vs. TCMO)			
Hsp 20	GCCAACGATGTCCAGAGATT CTGCCTCTCCTCGTGCTTAC	196	heat shock protein hsp20.1
odcdl	AAATGTCTGGGTGGAAGCAG GGGTGGAATCATCAGGTTGT	235	oxygen-dependent choline dehydrogenase-like
myr	GGAGACCGAGTGAAGACCTG GCTGTGGCATGAGCTAACAA	152	myrosinase 1
Upregulated genes (CAR vs. TCMO)			
pl	TGTTGTCGCTTTTCATCTGC AGTTTTTGAGCGCCGTATTG	207	peroxidase-like
LOC105842393	AAATGGCGCACAAATAGAGG CCAAGCTCTGCGTAAGGTTC	177	uncharacterized protein
ts	AAACCGAGCGTCCACTTATG TATTGTGATGGCAGCGGTAA	186	uncharacterized protein (tetraspanin family)
Housekeeping gene for normalization			
rp49	CAGGCGGTTCAAGGGTCAATAC TGCTGGGCTCTTTCCACGA	213	ribosomal protein 49

**Table 2:** Characteristics of silkworm strains used in the study.

Strain	Origin	Strain Classification	Cocoon	Cocoon Shell Percentage	Raw Silk Percentage
MO204	TCMO	Priority	Oval, white	22.92%	22.42%
DMMMSU119	TCMO	Poor performing	Peanut, white	21.81%	21.12%
LAT51	CAR	Priority	Peanut, white	21.57%	17.44%
ITA	CAR	Priority	Oval, yellow gold	20.27%	no data

Cocoon shell percentage: Ratio of the weight of silk shell to the weight of the whole cocoon; Raw silk percentage: Ratio of the weight of raw silk to the weight of the whole cocoon.

**Table 3:** Statistics of raw RNA-seq read sequences of *Bombyx mori* strains.

Sample	Read Count	% ≥ Q30	Mean Q Score	GC Content (%)
MO204-1	17,531,032	94.11	34.60	41
MO204-3	8,590,562	93.49	34.47	43
MO204-4	13,085,562	94.10	34.59	44
DMMMSU-0	11,406,238	92.68	34.29	46
DMMMSU-2	21,789,720	92.38	34.22	46
DMMMSU-4	15,464,914	91.51	34.04	46
LAT51-1	12,321,992	93.70	34.51	42
LAT51-2	22,767,422	93.91	34.56	41
LAT51-4	8,793,834	94.74	34.73	44
ITA-1	3,621,664	86.80	33.05	48
ITA-2	4,633,324	88.96	33.51	48
ITA-3	3106	65.36	28.55	75

**Table 4:** Contig statistics of the different transcriptome assemblies from *Bombyx mori* strains. Statistics generated from TrinityStats.pl.

	Trinity	Cufflinks	StringTie
Total Trinity “genes”	47,562	40,593	41,851
Total Trinity transcripts	69,948	40,593	41,851
Percent GC	38.12%	42.08%	41.66%
N10	3257	7944	7333
N20	2487	6108	5595
N30	1992	4987	4491
N40	1635	4126	3640
N50	1313	3414	2981
Median contig length	468	1738	1226
Average contig length	800.21	2305.35	1789.3

**Table 5:** DETONATE scores of the different *Bombyx mori* transcriptome assemblies.

Assembly	RSEM-Eval	Nucleotide F1 (Unweighted)	Contig F1 (Unweighted)	Weighted k-Mer Recall	k-Mer Compression Score
Trinity	−8.35 × 10^9^	0.38019	0.00239295	0.678414	−7.32371
Cufflinks	−1.34 × 10^10^	0.682955	0.690038	0.545637	−22.5079
StringTie	−1.55 × 10^10^	0.775391	0.677147	0.545727	−17.3472

**Table 6:** Summary of select top DEGs between different *Bombyx mori* strains (CAR vs. TCMO).

Downregulated Genes (CAR vs. TCMO)				
Gene ID	log_2_FC	*p*_adj_	Protein ID	Function
LOC101740965	−12.8030	1.31 × 10^−20^	myrosinase 1 isoform X2	hydrolysis of glucosinolates, defense enzyme
Hsp20.1	−4.4182	7.45 × 10^−17^	heat shock protein hsp20.1	chaperone protein
LOC101739591	−4.4249	7.92 × 10^−20^	transient receptor potential channel pyrexia isoform X1	ion transport, contains ankyrin repeat
LOC101741105	−10.2153	5.85 × 10^−13^	myrosinase 1	hydrolysis of glucosinolates, defense enzyme
LOC101738036	−10.7102	2.66 × 10^−10^	serine protease inhibitor swm-1	serine protease inhibitor, trypsin inhibitor
LOC105842960	−3.8422	4.35 × 10^−15^	oxygen-dependent choline dehydrogenase-like	oxidoreductase activity, FAD/NAD(P)-binding
**Upregulated genes (CAR vs. TCMO)**				
**Gene ID**	**log_2_FC**	***p*_adj_**	**Protein ID**	**Function**
LOC101739033	5.7239	7.09 × 10^−16^	zinc finger protein 664	dimer formation, metal binding
LOC101740169	8.3899	1.81 × 10^−14^	uncharacterized protein LOC101740169	tetraspanin family, scaffolding, cell adhesion, proliferation
LOC101741524	6.9289	1.99 × 10^−12^	synaptic vesicle glycoprotein 2C	sugar transporter
LOC105842878	3.3250	2.85 × 10^−9^	peroxidase-like	heme peroxidase, redox activity, oxidoreductase activity, defense enzyme
LOC119629954	7.5484	1.91 × 10^−8^	zinc finger BED domain-containing protein 4-like isoform X2	contains hAT dimerization domain, ribonuclease H-like superfamily: nucleotide metabolism and transport
LOC119630495	9.2422	5.08 × 10^−7^	uncharacterized protein LOC119630495	contains motifs: peptidase, Hsp70, TetR
LOC105842393	6.7307	1.61 × 10^−6^	uncharacterized protein LOC105842393	contains motifs: DUF5641 (pfam18701), unknown function, found in retrotransposons
Defensin	2.2251	5.08 × 10^−7^	defensin like protein 2 precursor	contains signal peptide

## Data Availability

The RNA-seq dataset generated in this study has been deposited in the National Center for Biotechnology Information (NCBI) Gene Expression Omnibus [45] accessible through GEO Series Accession Number GSE184152.

## Supplementary Materials

The following are available online at https://www.mdpi.com/article/10.3390/insects13080669/s1, Table S1: DEGs between *B. mori* CAR and TCMO strains within the *p*-adj threshold, Table S2: GO terms enriched among upregulated genes in the silk glands of selected *B. mori* strains from CAR, Table S3: GO terms enriched among downregulated genes in the silk glands of selected *B. mori* strains from CAR.
