# Supplementary material for: Global Profiling of Genes Expressed in the Silk Glands of Philippine-Reared Mulberry Silkworms (Bombyx mori)
Source: Insects. 2022 Jul 24;13(8):669. doi: 10.3390/insects13080669 (PMC9329738; doi:10.3390/insects13080669)
Supplement: Supplementary file 1 [file insects-13-00669-s001.zip › insects-1808631-supplementary.pdf]

Table S1. DEGs between *Bombyx mori* CAR and TCMO strains within the p-adj threshold.

Gene ID	Normalized mean exp	log_2 FoldChange	p_{adj}	nr protein ID	Description
LOC101740965	436.3777	-12.8030	1.31E-20	XP_021203658.1	myrosinase 1 isoform X2
LOC101739591	85.9320	-4.4249	7.9232E-20	XP_004926185.2	transient receptor potential channel pyrexia isoform X1
Hsp20.1	292.2198	-4.4182	7.45747E-17	NP_001036941.1	heat shock protein hsp20.1
LOC101739033	79.1756	5.7239	7.08597E-16	XP_004928701.1	zinc finger protein 664
LOC105842960	215.2818	-3.8422	4.3454E-15	XP_012552891.2	oxygen-dependent choline dehydrogenase-like
LOC101741132	155.2555	-4.9429	6.18552E-15	XP_037876863.1	uncharacterized protein LOC101741132
LOC101740169	134.2197	8.3899	1.81336E-14	XP_004921688.3	uncharacterized protein LOC101740169
LOC101745002	272.0163	-3.4352	1.11654E-13	XP_004931443.1	uncharacterized protein LOC101745002
LOC101743834	30.4942	-4.5650	4.97996E-13	XP_037873172.1	alkaline phosphatase, tissue-nonspecific isozyme isoform X1
LOC101741105	66.9775	-10.2153	5.845E-13	XP_021203655.1	myrosinase 1
LOC101738171	162.2757	-2.0042	6.52152E-13	XP_004927331.1	SOSS complex subunit B homolog
LOC101741524	100.6655	6.9289	1.98992E-12	XP_004926609.1	synaptic vesicle glycoprotein 2C
LOC101742796	649.1628	3.5420	4.13955E-12	XP_004929128.1	carbonyl reductase [NADPH] 3
LOC101740432	104.2471	3.0572	4.37612E-12	XP_004933592.1	homocysteine-responsive endoplasmic reticulum-resident ubiquitin-like domain member 2 protein
LOC101746050	8256.2203	-5.2850	2.99109E-11	NA	uncharacterized LOC101746050
LOC100101163	49.3780	-3.2183	1.84277E-10	NP_001093089.1	methyltransferase [Bombyx mori]
LOC101738036	76.9355	-10.7102	2.66044E-10	XP_004924398.1	serine protease inhibitor swm-1
LOC119629873	71.3362	-5.3808	6.24984E-10	XP_037873295.1	uncharacterized protein LOC119629873
LOC101746265	42.5051	-4.0678	1.15354E-09	XP_037869847.1	integral membrane protein GPR155 isoform X2
LOC105842878	2296.9477	3.3250	2.84903E-09	XP_037874818.1	peroxidase-like
6pgl	48.4983	3.6675	4.63226E-09	NP_001091839.1	6-phosphogluconolactonase
LOC101736392	957.5713	-3.2824	1.02626E-08	XP_021209119.1	L-sorbose 1-dehydrogenase
LOC119629954	186.8266	7.5484	1.91075E-08	XP_037873483.1	zinc finger BED domain-containing protein 4-like isoform X2
LOC101739276	76.9759	6.1927	2.16059E-08	XP_037870249.1	glucose dehydrogenase [FAD, quinone]
LOC101746864	62.8409	-3.1714	3.25936E-08	XP_012549821.2	protein Aster-B isoform X2
LOC119629130	71.6572	-4.1325	3.70854E-08	XP_037869956.1	uncharacterized protein LOC119629130
LOC732895	58.3223	3.7175	8.47329E-08	NP_001040361.1	heme oxygenase

LOC101743364	48.8056	-2.6748	3.22564E-07	XP_037871715.1	uncharacterized protein LOC101743364 isoform X1
LOC101744567	20.9682	-8.8363	5.08256E-07	XP_012548358.1	neuroligin-1
Defensin	342.2128	2.2251	5.08256E-07	NP_001128677.1	defensin like protein 2 precursor
LOC119630495	28.3819	9.2422	5.08256E-07	XP_037876003.1	uncharacterized protein LOC119630495 isoform X1
LOC101744852	106.5376	7.2630	6.16541E-07	XP_037876651.1	cytochrome P450 4V2 isoform X4
LOC101737305	37.1197	3.9218	7.11968E-07	XP_004927603.2	cytochrome P450 4C1
LOC119629803	128.9255	7.6255	7.86642E-07	XP_037872937.1	zinc finger BED domain-containing protein 4-like
LOC119629818	127.9309	7.6242	8.07675E-07	XP_037873002.1	zinc finger BED domain-containing protein 4-like
LOC119629972	88.1535	5.2284	9.40984E-07	XP_037873662.1	uncharacterized protein LOC119629972
LOC105842393	62.8641	6.7307	1.61056E-06	XP_037868479.1	uncharacterized protein LOC105842393
LOC101745011	19.5005	3.5491	1.83925E-06	XP_021208151.2	complexin isoform X2
LOC101741044	159.2426	-1.8129	2.43435E-06	XP_004922247.3	uncharacterized protein LOC101741044 isoform X2
UGT48C1	178.9960	-7.9885	3.60103E-06	XP_021203370.2	UDP-glycosyltransferase UGT48C1 isoform X1
LOC732890	56.6049	2.9106	5.49555E-06	NP_001040356.1	cytidylate kinase
LOC105841524	48.0799	-4.5150	6.2951E-06	XP_021203172.2	uncharacterized protein LOC105841524 isoform X2
LOC101736673	138.5163	3.5415	6.2951E-06	XP_004928770.2	collagen alpha-1(I) chain
LOC119629287	55.3664	5.8955	7.62398E-06	XP_037870519.1	uncharacterized protein LOC119629287
LOC101735767	100.4671	1.7779	7.9117E-06	XP_037876497.1	methionine--tRNA ligase, mitochondrial
LOC101746671	149.6756	5.5972	1.46576E-05	XP_004930015.2	uncharacterized protein LOC101746671
LOC101738200	373.9220	-6.3058	1.46576E-05	XP_012544353.2	fibroin heavy chain-like isoform X1
LOC101735908	22.6089	-3.5650	1.46576E-05	XP_037873557.1	alpha-protein kinase 1
LOC101744531	82.6066	-2.3253	1.62158E-05	XP_004932523.1	charged multivesicular body protein 6-A
LOC101738292	681.7750	2.4229	1.68767E-05	XP_004922915.1	5-aminolevulinate synthase, nonspecific, mitochondrial
LOC119629772	40.7115	-4.8036	1.68767E-05	XP_037872819.1	uncharacterized protein LOC119629772
LOC119628469	39.2529	-4.5224	1.72227E-05	XP_037866660.1	uncharacterized protein LOC119628469
LOC101739203	77.1415	3.2882	2.2298E-05	XP_004921596.1	apolipoprotein D
LOC101737676	1872.7443	1.4583	2.23613E-05	XP_004924612.2	esterase FE4
LOC101740369	24.7646	-2.9164	2.32599E-05	XP_037874125.1	RNA-binding protein fusilli isoform X2
LOC110386615	50.6045	-4.0303	2.73796E-05	XP_037874399.1	uncharacterized protein LOC110386615
LOC101737374	28.8365	-8.1384	4.79553E-05	XP_021207354.1	cytochrome P450 4C1 isoform X1
LOC101738942	1500.2417	1.7097	5.03961E-05	XP_021206280.1	luciferin sulfotransferase isoform X2
LOC100141438	29.0592	-3.1726	5.03961E-05	XP_037873044.1	RAC serine/threonine-protein kinase

LOC119628846	29.3121	-6.5021	5.36575E-05	XP_037868561.1	uncharacterized protein LOC119628846
LOC101745355	106.1541	1.5507	5.71438E-05	XP_004921704.1	probable methylthioribulose-1-phosphate dehydratase
LOC110384718	88.9101	-4.3995	6.29288E-05	XP_021202481.1	fungal protease inhibitor F-like
LOC101739257	47.4801	-1.8892	6.35415E-05	XP_037874882.1	exocyst complex component 4
LOC692953	692.4078	1.9867	6.59383E-05	NP_001040258.1	mitochondrial matrix protein p33
LOC119630131	61.2260	5.3064	7.45402E-05	XP_037874548.1	uncharacterized protein LOC119630131
LOC101743457	87.2210	-3.2798	7.85846E-05	XP_037875239.1	phosphoglycolate phosphatase 1A, chloroplastic isoform X2
LOC110384866	48.8552	4.8804	8.1652E-05	XP_037876336.1	uncharacterized protein LOC110384866
LOC101743996	649.6855	-3.4249	8.18676E-05	XP_004930083.1	mitochondrial enolase superfamily member 1
LOC101737570	249.9943	1.5609	8.18676E-05	XP_004923805.1	uncharacterized protein LOC101737570 isoform X2
LOC101743012	157.5805	-2.4119	8.18676E-05	XP_004922735.2	zinc finger protein 511
LOC101740590	21.8677	3.3215	8.18676E-05	XP_004926527.1	NADH-cytochrome b5 reductase-like
LOC101746778	22.6506	-3.3239	0.000114347	XP_004931758.1	uncharacterized protein LOC101746778
LOC101742375	26.9154	-6.5824	0.000117341	XP_004921964.1	intraflagellar transport protein 56 isoform X2
LOC110386896	23.0589	-2.6427	0.000180562	XP_037871766.1	zinc finger protein 679-like
LOC101736092	94.8939	-4.8740	0.000204722	XP_021203605.1	sugar transporter ERD6-like 6
LOC101738097	85.6395	-1.9410	0.000213571	XP_021207458.1	mucin-5AC isoform X1
LOC733096	65.5349	1.5271	0.000243971	NP_001040508.1	DCMP deaminase
LOC119629365	20.2491	-6.7152	0.000264902	XP_037870961.1	piggyBac transposable element-derived protein 3-like
LOC101742019	120.6296	1.9747	0.000299259	XP_037870489.1	medium-chain acyl-CoA ligase ACSF2, mitochondrial isoform X1
LOC101737615	58.0652	-1.7416	0.000307651	XP_004921584.1	B-cell CLL/lymphoma 7 protein family member A
LOC101741708	89.9756	2.3207	0.000344133	XP_012544065.1	b(0,+)-type amino acid transporter 1 isoform X2
LOC101745433	510.7035	-1.1336	0.000397207	XP_037866532.1	endochitinase A
LOC101743020	27.1457	-2.4883	0.00040381	XP_004924866.1	allantoinase isoform X2
LOC119630755	45.0724	6.1943	0.000418409	XP_037877167.1	uncharacterized protein LOC119630755
LOC110386515	38.5069	-4.1036	0.000419744	NA	uncharacterized LOC110386515
LOC101745700	18.2071	-3.5372	0.000440811	XP_004922195.2	zinc finger protein 235-like
LOC110385639	11.0526	-6.5652	0.000440811	NA	uncharacterized LOC110385639
LOC119629474	25.0925	-5.4229	0.000450222	XP_037871247.1	piggyBac transposable element-derived protein 3-like
LOC101736158	103.1197	1.8942	0.000450222	XP_004931999.1	nuclear transcription factor Y subunit B-1 isoform X1
LOC101746910	7070.8232	2.8209	0.000457546	XP_012548096.1	glucose dehydrogenase [FAD, quinone]
GSTz2	123.6960	-2.2645	0.000460761	NP_001040453.1	glutathione S-transferae zeta 2

LOC101747201	15.6919	5.7137	0.0006104	XP_037867172.1	15-hydroxyprostaglandin dehydrogenase [NAD(+)]-like
LOC119629696	32.1141	-4.5968	0.000645091	XP_037872383.1	uncharacterized protein LOC119629696
LOC101739623	59.8369	-1.5139	0.000743006	XP_012551644.2	E3 ubiquitin-protein ligase MGRN1 isoform X2
bcpi	1471.3920	1.1843	0.00080402	NP_001037057.1	BCP inhibitor precursor
LOC101744314	12.2742	4.8557	0.000848049	XP_037874390.1	myogenesis-regulating glycosidase isoform X5
Hr3	36.9941	2.6212	0.000872001	NP_001037012.1	hormone receptor 3
LOC101742888	24.7841	-2.2218	0.000925236	XP_004927023.1	probable RNA methyltransferase CG11342
LOC101736515	185.9575	3.6139	0.000952687	XP_004923796.2	centromere/kinetochore protein zw10
Cbp	2164.0748	-2.5407	0.001053862	NP_001036998.2	carotenoid-binding protein isoform 1
LOC101745024	110.4557	-1.4067	0.001083378	XP_012553114.1	serine/threonine-protein phosphatase 5
LOC101741933	9.2083	-6.5633	0.001091497	XP_004931726.3	uncharacterized protein LOC101741933
LOC101735447	11944.5239	-3.7859	0.001236911	XP_012550392.2	low molecular mass 30 kDa lipoprotein 19G1-like isoform X1
serpin-3	46.7019	-2.8853	0.001409995	NP_001139723.1	serine protease inhibitor 32 precursor
LOC101735499	20.6371	2.6719	0.001513601	XP_037869262.1	nuclear pore complex protein Nup154
LOC101737540	483.2710	-1.1185	0.001538896	XP_021203782.2	uncharacterized protein LOC101737540
Fps	523.7709	0.9780	0.001582667	NP_001036889.1	farnesyl pyrophosphate syntase
LOC119629588	15.2652	3.0041	0.001582801	XP_037871813.1	uncharacterized protein LOC119629588 isoform X2
LOC101737046	55.3370	1.4228	0.00168536	XP_004922840.1	GDP-D-glucose phosphorylase 1
LOC101746460	103.7242	1.7427	0.001685772	XP_037871156.1	glucosamine-6-phosphate isomerase isoform X1
LOC101743840	348.4453	-3.7661	0.001685772	XP_021205460.1	poxin isoform X1
LOC101740006	14.0182	-4.8865	0.001685772	XP_004927006.2	muskelin isoform X1
Ser3	45997.7312	-3.2035	0.001798348	NP_001108116.1	sericin 3 precursor
LOC101737798	580.9310	-1.4028	0.001798348	XP_037876890.1	monosaccharide-sensing protein 2-like
LOC101738622	54.3368	-1.7508	0.001837383	XP_004929858.1	protein zer-1 homolog
LOC101741066	268.5588	3.8200	0.001837383	XP_037872403.1	farnesol dehydrogenase isoform X2
LOC101738233	20.1667	3.6308	0.001837383	XP_021207797.1	sodium-independent sulfate anion transporter
LOC692390	1070.7259	1.5822	0.001946265	NP_001036850.1	cathepsin B precursor
LOC101736553	10.9803	-6.7070	0.002049542	XP_021208619.1	pancreatic lipase-related protein 2 isoform X1
LOC101737629	197.4987	-1.4563	0.002072972	XP_037877610.1	protein transport protein Sec31A isoform X1
Lp-c12	11734.4947	-3.9418	0.002088225	NP_001095196.1	low molecular 30 kDa lipoprotein PBMHP-12 precursor
LOC101745090	69.4287	-1.7762	0.002109124	XP_004928155.1	uncharacterized protein LOC101745090
LOC101738851	35.8867	2.0885	0.002109124	XP_037874493.1	Bloom syndrome protein homolog isoform X4

LOC101744388	133.2889	1.7375	0.002357683	XP_004933079.2	heparanase
LOC119629464	11.1358	-5.8035	0.002384697	NA	uncharacterized LOC119629464
LOC101739542	154.2530	1.1554	0.002501082	XP_037874453.1	E3 ubiquitin-protein ligase RNF126 isoform X2
LOC101736715	31.3738	-5.3239	0.002611968	XP_004927192.1	uncharacterized protein LOC101736715 isoform X1
LOC105842712	17.7374	-4.4995	0.00312289	XP_012552399.1	piggyBac transposable element-derived protein 3
LOC101747147	9.1078	5.8310	0.003361305	XP_004932959.1	sodium-coupled monocarboxylate transporter 2 isoform X1
LOC101740041	67.5965	-1.2152	0.003385278	XP_037871472.1	rho GTPase-activating protein 26 isoform X2
LOC101741909	236.4112	-1.8386	0.003515856	XP_037867532.1	zonadhesin isoform X5
LOC101738370	35.0129	1.9059	0.003681913	XP_037875792.1	dipeptidyl peptidase 9 isoform X1
LOC101746588	56.6261	-2.2034	0.003785429	XP_004931352.1	copper homeostasis protein cutC homolog isoform X2
NGR-B3	93.5326	-3.0348	0.003785429	NP_001127734.1	neuropeptide receptor B3 precursor
LOC101746342	208.8797	1.8174	0.003785429	XP_037876002.1	probable C-mannosyltransferase DPY19L1 isoform X4
LOC101736769	350.2724	0.9123	0.003806654	XP_037867014.1	peroxidasin isoform X2
Cyp6ab5	12.2075	4.7385	0.003806654	NP_001104007.1	cytochrome P450, family 6, subfamily ab, polypeptide 5
CPR100	25.6407	-4.2721	0.003835028	NP_001166666.1	cuticular protein RR-2 motif 100 precursor
LOC101745301	17.9101	5.5845	0.004136918	XP_004931827.1	SET domain-containing protein SmydA-8
LOC101736683	29.2235	-2.6711	0.004219006	XP_012550355.1	uncharacterized protein LOC101736683 isoform X1
LOC101735438	28.8171	1.7773	0.004284124	XP_012551683.1	erythroid differentiation-related factor 1
LOC119629020	15.0173	4.3707	0.004318845	NA	uncharacterized LOC119629020
LOC101740099	86.3404	1.5094	0.004318845	XP_037873937.1	balbiani ring protein 3 isoform X6
LOC101737414	862.7412	-1.8526	0.004393456	XP_004932559.2	dihydropyrimidine dehydrogenase [NADP(+)]
LOC110385229	47.5588	3.1399	0.004393456	NA	uncharacterized LOC110385229
LOC101738447	62.7279	-2.3555	0.004552091	XP_037873274.1	heme transporter hrg1-A
LOC101739299	330.8936	-1.7202	0.004912662	XP_037874377.1	probable isocitrate dehydrogenase [NAD] subunit alpha, mitochondrial isoform X2
LOC101745377	402.7614	-4.1387	0.005254193	XP_012545734.2	uncharacterized protein LOC101745377 isoform X1
LOC101737347	497.0754	6.9329	0.005445737	XP_004926654.1	calexcitin-2
CPG18	107.1604	-4.3882	0.00557497	NP_001166786.1	cuticular protein glycine-rich 18 precursor
LOC119630067	1320.5437	-4.3720	0.005592678	XP_037874089.1	leech-derived tryptase inhibitor C-like
LOC101741441	632.7040	1.3210	0.005828885	XP_004928837.2	protein windbeutel
LOC101746346	20.1012	4.1215	0.005882442	XP_012548107.2	phospholipase A2 isoform X1
LOC101735657	48.1814	1.8607	0.006002377	XP_037866431.1	retinol-binding protein pinta isoform X1

LOC101736654	105.2267	1.1135	0.006011422	XP_004924197.1	PCNA-associated factor
LOC101741202	14.7489	3.3288	0.006095487	XP_004924790.1	protein spaetzle 5
LOC119630233	27.7309	4.2403	0.006095487	XP_037874852.1	uncharacterized protein LOC119630233
LOC101744339	102.1022	-1.1300	0.006148767	XP_004932094.1	general transcription factor IIH subunit 2
LOC101738392	72.6024	1.5487	0.006640209	XP_004926995.1	probable protein BRICK1-B
Pi-t1	43.2140	6.8978	0.006683902	XP_012545178.1	inorganic phosphate transporter 1 isoform X1
LOC101745811	79.1749	-3.0516	0.006683902	XP_037869338.1	receptor-type guanylate cyclase Gyc76C
BGRP1	93.3594	-4.5930	0.006683902	NP_001036840.1	beta-1,3-glucan-binding protein precursor
serpin-21	16.6777	2.4629	0.006683902	XP_012547718.2	serine protease inhibitor 21 isoform X2
LOC732939	144.0499	-1.3130	0.006683902	NP_001040403.1	protein tyrosine phosphatase
LOC119629761	96.8824	1.9886	0.006776911	NA	uncharacterized LOC119629761
LOC101735802	45.6385	-1.6993	0.007042	XP_037877676.1	nuclear factor of activated T-cells 5 isoform X3
LOC119630597	54.7109	2.3391	0.007184144	XP_037876439.1	putative nuclease HARBI1
LOC101736055	49.4370	1.5404	0.00721305	XP_004929249.1	alpha-tocopherol transfer protein-like
LOC101742946	48.4280	1.3929	0.007618459	XP_037866929.1	U2 snRNP-associated SURP motif-containing protein isoform X1
Pbanr	43.1013	1.9007	0.007644961	NP_001036977.1	pheromone biosynthesis activating neuropeptide receptor
LOC101736451	13.0569	-3.6761	0.007951096	XP_012548466.2	adenylate kinase isoenzyme 5 isoform X1
LOC101742504	243.4709	1.2674	0.008118876	XP_004928639.1	deoxynucleotidyltransferase terminal-interacting protein 2
LOC101747035	12.6918	-2.7384	0.008118876	XP_037876045.1	uncharacterized protein LOC101747035 isoform X3
aif	55.6195	-4.9982	0.00817319	NP_001189462.1	apoptosis-inducing factor
LOC101745380	37.0384	-1.6647	0.008305924	XP_004926344.1	putative Dol-P-Glc:Glc(2)Man(9)GlcNAc(2)-PP-Dol alpha-1,2-glucosyltransferase
LOC110385006	14.6201	-3.7004	0.008340161	NA	uncharacterized LOC110385006
LOC101737789	49.5051	-1.5932	0.008466473	XP_004930829.2	zinc finger protein 1
LOC101738327	163.7102	1.1017	0.00848003	XP_004922571.1	peroxisome biogenesis factor 10
LOC110385781	16.6583	-2.2657	0.008480489	XP_021205836.1	zinc finger protein 506
LOC693069	1014.8224	0.8459	0.00872048	XP_012551823.1	protein kinase c inhibitor isoform X1
LOC101743935	18.1234	-5.7499	0.008998049	XP_037867726.1	synaptic vesicle 2-related protein
LOC101744645	41.3446	-2.1529	0.008998049	XP_021203165.1	uncharacterized protein LOC101744645 isoform X3
LOC119630438	19.1528	2.4834	0.008998049	NA	uncharacterized LOC119630438
LOC119631038	12.0177	-5.5486	0.008998049	XP_037877480.1	uncharacterized protein LOC119631038
ae25	1157.4771	0.8128	0.009061947	NP_001121784.1	alpha-esterase 25

LOC101745270	49.2460	-1.4138	0.009117792	XP_004922192.1	gastrula zinc finger protein xLCGF3.1-like
LOC101740700	313.6924	-1.0781	0.009117792	XP_004933459.1	threonine--tRNA ligase 1, cytoplasmic isoform X2
LOC101746572	124.2922	1.1811	0.00930581	XP_004927517.1	venom allergen 5
LOC101743783	125.4666	-3.5698	0.009546241	XP_004926544.2	alpha-aminoadipic semialdehyde synthase, mitochondrial
LOC110385255	81.5810	1.9876	0.010048603	NA	uncharacterized LOC110385255
LOC119630363	48.4674	-3.1852	0.010074067	XP_037875446.1	uncharacterized protein LOC119630363
LOC101739130	47.1297	1.5048	0.010103089	XP_012546966.1	uncharacterized protein LOC101739130 isoform X2
LOC119630310	2725.0231	5.1326	0.011026139	XP_037875176.1	probable protein phosphatase DDB_G0282105
LOC101741156	54.9691	1.8039	0.011026139	XP_004926607.1	synaptic vesicle 2-related protein isoform X1
LOC119630039	7.9873	4.8859	0.011026139	XP_037873922.1	uncharacterized protein LOC119630039 isoform X2
LOC119630340	55.1884	2.6400	0.011282782	NA	uncharacterized LOC119630340
LOC101747227	112.1781	1.7345	0.01142055	XP_037874831.1	cystinosin homolog isoform X2
LOC119630069	114.2214	1.5548	0.011521776	NA	uncharacterized LOC119630069
LOC101740008	17.0264	2.7722	0.011531323	XP_037875735.1	activity-regulated cytoskeleton associated protein 2 isoform X2
LOC101737319	40.7963	-1.5975	0.011854464	XP_037866795.1	arfGAP with SH3 domain, ANK repeat and PH domain-containing protein
LOC101744002	109.9652	-2.1056	0.012411105	XP_037874975.1	uncharacterized protein LOC101744002 isoform X2
LOC692743	25.2986	1.6365	0.01242231	NP_001104817.1	hemocytin
Anxb13	55.2056	-3.6199	0.01242231	NP_001036881.1	annexin B13
LOC119629744	3782.7543	-3.8704	0.013938696	NA	uncharacterized LOC119629744
LOC119630552	468.6001	6.8415	0.013953725	XP_037876282.1	uncharacterized protein LOC119630552
LOC119628590	10.0443	2.9698	0.013953725	NA	U6 spliceosomal RNA
LOC101738067	18.1913	-3.7450	0.0144057	XP_004922569.1	uncharacterized protein LOC101738067 isoform X1
LOC101742140	270.1456	-0.9117	0.014588048	XP_004922106.1	protein TRC8 homolog
LOC101746901	22.1271	2.4363	0.014772403	XP_004926641.1	uncharacterized protein LOC101746901
LOC119629473	35.3614	-2.5051	0.015229363	XP_037871245.1	uncharacterized protein LOC119629473 isoform X1
LOC119630219	115.0564	1.5478	0.015385681	NA	uncharacterized LOC119630219
LOC101747054	1038.1596	-1.9193	0.015548832	XP_037872304.1	cytosolic 10-formyltetrahydrofolate dehydrogenase
LOC105842192	145.1087	-2.7541	0.015693241	XP_037866765.1	E3 SUMO-protein ligase ZBED1-like isoform X1
LOC119628805	117.9789	-2.6191	0.015777985	XP_037868454.1	E3 SUMO-protein ligase ZBED1-like
LOC101736941	17.2266	2.3697	0.015777985	XP_037870401.1	DDB1- and CUL4-associated factor 7
LOC100862837	26.8426	2.2233	0.015823702	NP_001243987.1	laminin beta-2 chain

LOC119629466	124.9122	-2.6771	0.015896725	XP_037871213.1	E3 SUMO-protein ligase ZBED1-like
LOC101740637	9.2388	6.3410	0.016072467	XP_037870853.1	organic cation transporter protein
LOC105842089	1306.9154	-1.2964	0.016325196	NA	uncharacterized LOC105842089
LOC101747097	89.3299	1.6019	0.016501924	XP_037874112.1	piggyBac transposable element-derived protein 4 isoform X1
LOC101744559	52.9519	2.7408	0.016657549	XP_037872288.1	4-coumarate--CoA ligase 1
LOC101742496	359.4151	-2.5713	0.016912277	XP_004926880.2	lysosomal acid glucosylceramidase isoform X1
LOC101740833	34.9767	2.7517	0.017975495	XP_037867180.1	uncharacterized protein LOC101740833 isoform X2
LOC119629479	52.9033	2.5507	0.017975495	NA	uncharacterized LOC119629479
LOC101738547	27.8274	5.2226	0.018075138	XP_012550588.2	uncharacterized protein LOC101738547
LOC101741173	102.1429	-1.1233	0.018075138	XP_021206845.1	probable phosphorylase b kinase regulatory subunit beta isoform X5
LOC105842032	10.1780	-3.9996	0.018762185	NA	uncharacterized LOC105842032
LOC692961	13.8859	2.7570	0.01890377	NP_001040266.1	peroxisomal membrane anchor protein
LOC119629492	116.7996	1.5473	0.018982016	NA	uncharacterized LOC119629492
LOC101740686	118.4307	1.3334	0.019666643	NP_001091795.2	uncharacterized protein LOC778500
LOC101746454	20.1610	2.2083	0.019976797	XP_004933013.1	uncharacterized protein LOC101746454
LOC101738529	343.2288	1.0623	0.020211768	XP_004926437.1	putative riboflavin kinase
LOC692641	70.1263	1.2092	0.020317288	XP_037870667.1	hemolin-interacting protein isoform X1
LOC692889	242.5401	-2.6741	0.020399564	NP_001040198.1	mitochondrial aldehyde dehydrogenase
LOC119630931	40.8039	-2.5337	0.020399564	XP_037877398.1	uncharacterized protein LOC119630931 isoform X1
LOC101740778	34.3696	-2.1800	0.020425177	XP_012545410.1	solute carrier family 25 member 40
LOC119629041	44.1530	2.6111	0.020425177	NA	uncharacterized LOC119629041
Jheh-lp2	706.4487	2.0057	0.020425177	XP_012551156.1	juvenile hormone epoxide hydrolase-like protein 2 isoform X1
LOC119630443	127.9378	-2.6359	0.020425177	XP_037875845.1	E3 SUMO-protein ligase ZBED1-like isoform X1
LOC101744175	23.5616	-5.2183	0.020483839	XP_004926335.2	uncharacterized protein LOC101744175
LOC119630429	40.4766	-2.5092	0.020483839	XP_037875753.1	uncharacterized protein LOC119630429 isoform X1
LOC110386692	18.3815	2.2110	0.020483839	XP_037876528.1	putative nuclease HARBI1
LOC101746322	183.5695	-3.5909	0.021111605	XP_004923446.1	uncharacterized protein LOC101746322
LOC101745500	37.1421	-6.3024	0.021314753	XP_004933911.2	heat shock protein 68
LOC101745977	12.2182	2.9233	0.021475	XP_037869837.1	dynein intermediate chain 3, ciliary isoform X1
LOC101735832	70.7824	-5.2025	0.022260651	XP_037876874.1	heat shock protein 68
LOC119630910	40.4831	-2.4932	0.022266341	XP_037877370.1	uncharacterized protein LOC119630910 isoform X2

LOC119628546	40.3348	-2.4932	0.022376714	XP_037867082.1	uncharacterized protein LOC119628546 isoform X1
LOC101743061	16.6514	4.5251	0.022376714	XP_021206117.1	coiled-coil domain-containing protein 112 isoform X2
LOC101738817	89.8397	-1.2855	0.022487624	XP_004931785.1	fatty-acid amide hydrolase 2-B
LOC101740925	16.7327	3.0227	0.022961868	XP_004927860.1	LIM domain only protein 3 isoform X1
LOC110386138	7.0539	6.2283	0.023410754	XP_037874038.1	microvitellogenin
CPG16	95.7801	-5.0352	0.023447535	NP_001161189.1	cuticular protein glycine-rich 16 precursor
LOC101740975	6.8303	5.7860	0.023698478	XP_037866483.1	protein eyes shut
Sec61alpha	7714.9013	-0.5131	0.024175031	NP_001037628.1	transport protein Sec61 alpha subunit
LOC101744109	7.2015	-5.4583	0.025007568	XP_004923010.1	thioredoxin domain-containing protein 2
LOC101747044	209.3939	-1.0220	0.025497684	XP_012548413.1	calcium channel flower isoform X2
LOC101740701	11.0045	-2.3619	0.025798396	XP_037877166.1	uncharacterized protein LOC101740701 isoform X2
LOC119630724	101.9508	-2.7804	0.025798396	XP_037877017.1	E3 SUMO-protein ligase ZBED1-like
LOC101745785	116.6687	1.1430	0.026152852	XP_037868754.1	nostrin isoform X1
LOC101737191	268.0840	2.1530	0.026152852	XP_004924201.1	adenylyltransferase and sulfurtransferase MOCS3
LOC119629754	49.2189	2.0778	0.026152852	XP_037872680.1	putative nuclease HARBI1
LOC733093	169.0379	-6.1399	0.026152852	NP_001296526.1	heat shock protein 70 A1
LOC101743121	46.6809	1.5764	0.026511527	XP_004928141.1	mitochondrial 2-oxodicarboxylate carrier
LOC101737917	98.1441	-4.5415	0.02661084	XP_004928608.1	argininosuccinate synthase
UGT40A1	264.7748	1.8292	0.027758128	XP_021207027.1	phenol UDP-glucosyltransferase isoform X1
LOC110385299	13.8684	-2.6829	0.027896303	NA	uncharacterized LOC110385299
LOC101739133	208.8506	-1.7621	0.028015126	XP_004928444.1	forkhead box protein N3
LOC101742155	131.4707	1.1755	0.028434771	XP_004925979.1	zinc transporter ZIP9
LOC119629396	10.2031	-2.8794	0.029397831	XP_037871010.1	uncharacterized protein LOC119629396 isoform X2
LOC101736618	21.6834	-4.5748	0.02961971	XP_021204275.1	uncharacterized protein LOC101736618
LOC101736556	19.1988	-6.1704	0.029669414	XP_004922836.1	uncharacterized protein LOC101736556
LOC119629469	120.2180	-2.6178	0.029763987	XP_037871217.1	E3 SUMO-protein ligase ZBED1-like isoform X2
LOC101745388	7.1318	-5.1283	0.029814053	XP_037870547.1	glucose dehydrogenase [FAD, quinone] isoform X1
LOC101745643	383.3770	1.4224	0.029814053	XP_004921842.1	H/ACA ribonucleoprotein complex subunit 3
LOC101737886	36.5251	1.7453	0.029837634	XP_004933304.1	dymeclin
LOC101737012	247.3246	-0.7885	0.030110158	XP_004923800.1	AP-1 complex subunit mu-1
LOC101744161	24.6037	-2.8076	0.030369332	XP_004934376.1	uncharacterized protein LOC101744161
LOC101737792	98398.9313	-1.6625	0.030369332	NA	uncharacterized LOC101737792

LOC101736258	10.2078	-2.8967	0.030369332	XP_037872173.1	microprocessor complex subunit DGCR8
LOC119630704	6.3025	-7.0989	0.030369332	XP_037876982.1	facilitated trehalose transporter Tret1-like
LOC119629285	126.9290	-2.0555	0.030467521	XP_037870514.1	E3 SUMO-protein ligase ZBED1-like
LOC101740657	35.4777	1.3758	0.030629833	XP_004931797.2	EF-hand domain-containing protein D2 homolog
LOC119628831	94852.7065	-1.6467	0.030857697	XP_037868513.1	uncharacterized protein LOC119628831
LOC101736687	31.5429	1.4775	0.030857697	XP_012550842.1	integrator complex subunit 12
LOC119630586	374.6982	1.5903	0.030857697	XP_037876393.1	4-coumarate--CoA ligase 1-like
CPH43	98339.9145	-1.6542	0.031547246	NP_001166198.1	cuticular protein hypothetical 43
LOC110384846	55.6125	-5.7349	0.031547246	NA	uncharacterized LOC110384846
LOC101742998	9.6505	-5.7081	0.031569206	XP_037870167.1	laminin subunit alpha
LOC101739568	221.4114	1.0020	0.032012905	XP_012546333.1	zinc-type alcohol dehydrogenase-like protein SERP1785
LOC101735380	19.2599	-2.8609	0.032243371	XP_037871626.1	caytaxin
LOC105842250	23.8495	-3.9848	0.032243371	XP_037867444.1	acetyl-coenzyme A synthetase 1
LOC101746507	116.0722	-3.6588	0.032243371	XP_021205043.2	gelsolin
LOC101740822	38.4795	1.6476	0.032243371	XP_021204306.1	uncharacterized protein LOC101740822
Jheh2	230.7409	-1.7595	0.032522795	NP_001037201.2	juvenile hormone epoxide hydrolase precursor
AGPAT2	38.0930	-2.0268	0.032717968	NP_001266388.1	1-acylglycerol-3-phosphate O-acyltransferase 2
LOC119630684	69.8314	-2.4554	0.032984355	XP_037876866.1	uncharacterized protein LOC119630684
LOC101743176	39.5355	-3.8655	0.033662291	XP_004927095.1	uncharacterized protein LOC101743176
LOC119628848	74905.2360	-1.7668	0.034366636	NA	uncharacterized LOC119628848
LOC101744132	77.1404	-2.0090	0.034516246	NP_001299580.1	potassium voltage-gated channel subfamily H member 6-like
LOC105841738	67.5999	1.9196	0.034516246	XP_012546519.1	3-ketoacyl-CoA thiolase, mitochondrial
LOC101737321	33.4702	2.0387	0.034870313	XP_037870136.1	pre-mRNA-splicing factor ATP-dependent RNA helicase PRP16 isoform X3
Hsp23.7	122.4451	-1.8649	0.034895265	NP_001036942.1	heat shock protein hsp23.7 precursor
LOC119629460	56.5737	1.6072	0.035684467	XP_037871180.1	uncharacterized protein LOC119629460
LOC100101154	475.5711	1.2849	0.035684467	NP_001093080.1	ecdysteroid-regulated 16 kDa protein precursor
LOC119630428	142.8734	1.5937	0.035968369	NA	uncharacterized LOC119630428
LOC733048	49.5522	-1.4484	0.035968369	NP_001040505.1	vacuolar protein sorting 29
LOC101744960	173.9568	2.6379	0.036309368	XP_004931615.1	alanine--glyoxylate aminotransferase 2, mitochondrial
LOC119630068	63.3347	-2.3948	0.038078906	XP_037874105.1	uncharacterized protein LOC119630068
LOC101743813	30.4522	-4.5689	0.038166052	NP_001296544.1	uncharacterized LOC101743813 precursor

LOC101735683	17.4561	3.8061	0.038889751	XP_004922895.1	myophilin
LOC101737690	81.3622	-0.9384	0.038889751	XP_004928691.2	uncharacterized protein LOC101737690
LOC101735750	83.7756	5.7897	0.038889751	XP_021204987.2	uncharacterized protein LOC101735750
CPR102	17.8215	-4.6055	0.038889751	NP_001166664.1	cuticular protein RR-2 motif 102 precursor
LOC101737984	80.9916	-3.6911	0.039065285	XP_004923731.1	ommochrome-binding protein
LOC119628772	9.8880	2.4769	0.039927081	NA	U6 spliceosomal RNA
LOC119630793	10.0258	-5.8149	0.040298035	NA	uncharacterized LOC119630793
LOC692400	76.6567	-1.9076	0.040494838	NP_001036859.1	ecdysteroid-inducible angiotensin-converting enzyme-related gene product precursor
LOC105841915	24.6442	1.8240	0.040605825	NA	uncharacterized LOC105841915
LOC105841701	33.0616	1.9733	0.04158315	NA	uncharacterized LOC105841701
LOC101746721	11.9516	2.5696	0.041721027	XP_004929513.1	phospholipase A1
LOC105841833	6.8932	-3.1382	0.041721027	NA	uncharacterized LOC105841833
LOC101738281	39.7263	-2.5804	0.041899513	XP_037872876.1	solute carrier family 28 member 3
LOC101742436	92.0742	0.8814	0.041899513	XP_012552946.1	uncharacterized protein LOC101742436 isoform X2
LOC101745373	17.3787	-5.2325	0.04212556	XP_004924880.1	integrin beta-8 isoform X2
LOC100101225	62.9109	-1.4361	0.042560454	NP_001093318.1	methylated DNA-protein cysteine methyltransferase
LOC101740251	34.7831	-4.2956	0.043020783	XP_021207352.2	sialin
LOC101742931	73.9749	1.3046	0.043020783	XP_021203208.2	UDP-N-acetylglucosamine transferase subunit ALG13 homolog
LOC101742364	6.0161	-3.5493	0.04348108	XP_012550084.2	CDK5 and ABL1 enzyme substrate 2 isoform X2
LOC101741812	93.9780	-1.4497	0.043533408	XP_004927283.1	flap endonuclease 1
LOC101738998	45.2038	1.7580	0.044246903	NA	uncharacterized LOC101738998
LOC119629686	9.6060	-4.6288	0.044246903	XP_037872327.1	uncharacterized protein LOC119629686
LOC101744328	767.7122	0.8004	0.044413822	XP_037872347.1	alpha-tocopherol transfer protein-like
LOC101742342	130.5231	2.3235	0.04444487	XP_037876483.1	cytochrome P450 4V2
Mf-cpa	4.7859	4.8699	0.045663678	NP_001036933.1	molting fluid carboxypeptidase A precursor
LOC101738628	25.8428	4.0536	0.045663678	XP_004930880.1	maltase A1
LOC101746080	26.2357	-2.9412	0.045663678	XP_004921646.1	cilia- and flagella-associated protein 299 isoform X1
LOC110385599	108.0606	1.1225	0.045663678	NA	uncharacterized LOC110385599
LOC101740518	8.6175	4.4165	0.046112984	XP_004931946.1	elongation of very long chain fatty acids protein AAEL008004
LOC101741137	32.8835	-3.9105	0.047058755	XP_004922659.1	tryptophan 2,3-dioxygenase
LOC101745446	64.8051	0.9335	0.047239428	XP_004933009.1	TBC1 domain family member 7

LOC101736694	61.8507	1.3389	0.049263153	NA	uncharacterized LOC101736694
LOC101736870	54.5539	1.4139	0.049263153	XP_004921666.1	regulator of telomere elongation helicase 1 homolog isoform X1
LOC101738716	33.0626	-5.3256	0.049659187	XP_012548713.1	putative fatty acyl-CoA reductase CG5065 isoform X2
LOC110384702	8.6714	-4.6355	0.049659187	XP_021208475.1	uncharacterized protein LOC110384702
LOC101737397	61.7398	-3.3206	0.050294668	XP_004926295.1	uncharacterized protein LOC101737397
serpin-22	10205.4367	1.0866	0.050294668	NP_001139715.1	serine protease inhibitor 22 precursor
LOC101746063	232.5626	1.7900	0.053253987	XP_004930186.1	uncharacterized protein LOC101746063 isoform X1
raf	19.8223	3.9610	0.054871642	NP_001189459.1	raf kinase, effector of Ras
LOC119629983	10.7300	2.3425	0.054886606	NA	U6 spliceosomal RNA
Fln	22.2550	-5.9854	0.055195237	NP_001130045.1	flightin
LOC101739471	55.9416	-3.2999	0.055406675	XP_004930886.2	venom serine carboxypeptidase
LOC110384858	2254.7376	0.6030	0.056290067	XP_037868883.1	arylsulfatase B
LOC119628887	48.4605	1.6661	0.056290067	XP_037868835.1	piggyBac transposable element-derived protein 4-like isoform X2
LOC101739617	257.3515	1.0713	0.056290067	XP_004933048.1	exosome complex component CSL4
LOC101743258	25.3831	6.6577	0.056706356	XP_012545526.1	Bardet-Biedl syndrome 5 protein homolog
LOC101743076	391.4406	0.8008	0.056773096	XP_012546322.2	uncharacterized protein LOC101743076
Lsp-t	195.6942	-3.4947	0.057939724	NP_001036908.1	LSP-T protein precursor
LOC101741919	18.5975	-4.3027	0.058166335	XP_004928309.1	putative uncharacterized protein DDB_G0285869
LOC110384792	80.9967	1.4482	0.05973262	NA	uncharacterized LOC110384792
LOC101746321	88.4349	1.0450	0.060696179	XP_004922821.1	charged multivesicular body protein 2a
LOC101736114	801.6093	-1.0647	0.061263025	XP_021208433.1	2-amino-3-ketobutyrate coenzyme A ligase, mitochondrial
LOC101740198	31.3889	-6.1979	0.061916437	XP_021206262.1	uncharacterized protein LOC101740198
LOC119629478	49.6240	1.6225	0.061916437	XP_037871299.1	piggyBac transposable element-derived protein 4-like isoform X2
LOC101737724	49.1243	-2.5681	0.061916437	XP_021203566.1	digestive cysteine proteinase 1 isoform X1
LOC101746309	108.4360	-0.7310	0.061916437	XP_004931986.1	18S rRNA aminocarboxypropyltransferase
LOC101743953	40.4260	-4.2372	0.062346852	XP_021207905.1	uncharacterized protein LOC101743953 isoform X1
LOC101741119	2433.7513	0.9312	0.062445513	XP_021206281.2	sulfotransferase family cytosolic 1B member 1 isoform X1
LOC100141506	754.9367	-4.0529	0.062445513	NP_001112375.1	heat shock protein 25.4 precursor
LOC101745717	88.1543	0.8211	0.062445513	XP_004927177.1	reticulon-4-interacting protein 1, mitochondrial
LOC101738982	167.9906	0.8570	0.062445513	XP_021204562.1	mitochondrial import inner membrane translocase subunit TIM50-C isoform X2
LOC110385248	44.2267	2.0718	0.062445513	XP_037874908.1	uncharacterized protein LOC110385248

serpin-7	41.7396	-3.9145	0.062445513	NP_001139701.1	serine protease inhibitor 7 precursor
LOC119631020	5.1132	-4.7619	0.062445513	XP_037877468.1	uncharacterized protein LOC119631020
LOC778507	30.6882	1.3487	0.062980161	NP_001091802.1	uncharacterized protein LOC778507
LOC101744522	17.3507	4.6414	0.063386819	XP_037867150.1	monocarboxylate transporter 1 isoform X2
LOC119630620	6.1624	5.5786	0.063500166	XP_037876474.1	protein ALP1-like
LOC101739118	324.8750	0.7174	0.063500166	XP_004924837.1	dehydrogenase/reductase SDR family protein 7-like
LOC119629131	6.1624	5.5786	0.063500166	XP_037869966.1	protein ALP1-like
LOC101745849	147.2159	-2.7202	0.063500166	XP_037873941.1	ethanolaminephosphotransferase 1
LOC101744811	164.0180	-3.7033	0.063614802	XP_004930711.1	uncharacterized protein LOC101744811
LOC101739734	178.9284	1.3530	0.063769721	XP_037870571.1	solute carrier family 52, riboflavin transporter, member 3-B isoform X4
LOC692845	1100.1758	0.6842	0.064324713	NP_001040155.1	short-chain dehydrogenease/reductase-like
LOC101740948	144.8721	0.7438	0.066515927	XP_004932772.1	5-formyltetrahydrofolate cyclo-ligase
LOC101739774	140.6589	-3.3417	0.066515927	XP_037876345.1	phytanoyl-CoA dioxygenase, peroxisomal
LOC101742156	5.3986	-4.8872	0.066557292	XP_012545706.2	protein CBFA2T1 isoform X1
LOC101737198	41.3359	-1.5963	0.066683695	XP_037875997.1	dynactin subunit 1 isoform X8
LOC101736450	115.3880	-2.2105	0.067588306	XP_037872184.1	apyrase isoform X2
CPR104	11.0576	-4.8447	0.067963803	NP_001036854.1	cuticular protein RR-2 motif 104 precursor
LOC692685	447.0109	-4.3392	0.070097587	NP_001037199.1	promoting protein precursor
LOC101738081	320.7893	0.6492	0.070158443	XP_004925822.1	mitochondrial fission 1 protein
Hsp70	29.0997	-5.5801	0.070158443	XP_037876891.1	heat shock protein 70
LOC101745323	23.1913	5.0669	0.071048158	XP_004924743.1	proton-coupled folate transporter
LOC101743243	5.4293	3.2713	0.071048158	XP_004933071.1	phosphoacetylglucosamine mutase
LOC101740175	49.6814	1.2997	0.071107541	XP_021209097.1	leucine-rich repeat protein SHOC-2 isoform X3
LOC119629215	14.5767	-2.4969	0.071133038	NA	uncharacterized LOC119629215
LOC110386627	38.0830	-3.8295	0.071158248	NA	uncharacterized LOC110386627
LOC119629859	48.9398	1.6164	0.071167901	XP_037873204.1	piggyBac transposable element-derived protein 4-like isoform X2
LOC101742899	951.7628	0.5995	0.071212552	XP_004930162.1	probable very-long-chain enoyl-CoA reductase art-1
LOC101745023	1615.2361	-1.3013	0.071212552	XP_004922749.1	protein D2
LOC101743388	24.0588	-3.1354	0.071357311	XP_004923566.2	transmembrane protein 135 isoform X1
LOC101739163	141.6761	-0.8434	0.071357311	XP_037872058.1	filamin-A isoform X1
LOC100101183	67.6629	-3.2249	0.071357311	NP_001093281.1	vesicle amine transport protein

LOC101743420	556.1305	1.2307	0.071586642	XP_004931814.3	uncharacterized protein LOC101743420
LOC101736202	121.6214	0.7369	0.071667959	XP_004931273.1	3-oxoacyl-[acyl-carrier-protein] reductase FabG
LOC101739835	90.7839	0.9455	0.071667959	XP_004929469.1	sodium/hydrogen exchanger 9B2 isoform X7
LOC101735745	36.2416	1.2614	0.07175369	XP_004926778.1	F-box/LRR-repeat protein 20
LOC101740799	75.3352	-2.8651	0.071802323	XP_004930684.1	uncharacterized protein LOC101740799
LOC101745532	50.9017	1.8416	0.071960064	XP_004929426.1	probable ATP-dependent RNA helicase DDX28
LOC101746172	11.8843	3.6537	0.072093322	XP_004934209.2	uncharacterized protein LOC101746172
LOC101741494	36.5278	-1.4075	0.072352938	XP_012548024.1	uncharacterized protein LOC101741494
LOC101740372	443.9038	-1.4282	0.072352938	XP_012547588.1	visual system homeobox 2 isoform X1
ST2	162.0925	-1.5030	0.072352938	NP_001182632.1	sugar transporter protein 2
LOC101738343	100.0711	-2.2283	0.072973773	XP_004926302.1	lysozyme
LOC101746587	40.8760	-4.4829	0.072973773	XP_004931158.1	tetratricopeptide repeat protein 1
LOC101743242	436.5083	-2.7983	0.074178244	XP_037875112.1	putative uncharacterized protein DDB_G0282133
LOC101738115	29.2818	-1.3044	0.074419759	XP_004923663.1	6-phosphofructo-2-kinase/fructose-2,6-bisphosphatase isoform X4
LOC101736302	49.9014	-3.6508	0.074448534	XP_004922006.1	uncharacterized protein LOC101736302
LOC101745175	65.7524	-3.2503	0.074984383	XP_037876240.1	lopap
LOC101742613	462.7264	-3.2474	0.0767972	XP_004933832.1	uncharacterized protein LOC101742613
LOC101736858	147.1422	0.9471	0.077061798	XP_004932127.1	tRNA N(3)-methylcytidine methyltransferase METTL2 isoform X2
LOC101743405	63.3738	1.4237	0.077818155	XP_012547668.1	methyltransferase-like protein 25
LOC105842323	24.8943	-6.2212	0.078152199	XP_012550110.1	uncharacterized protein LOC105842323
LOC733001	57.3376	-3.2926	0.080535772	XP_037875581.1	serine proteinase-like protein isoform X1
LOC101736585	82.7758	0.9903	0.081277047	XP_004931276.1	short coiled-coil protein homolog
LOC105842145	43.4136	1.6323	0.081277047	NA	uncharacterized LOC105842145
LOC101735849	33.7318	1.6570	0.082131708	XP_012551913.1	4-coumarate--CoA ligase 1
NGR-B1	19.3961	-2.0683	0.08244969	XP_037868157.1	neuropeptide receptor B1 isoform X1
Cjhbp	96.0747	1.2326	0.082776009	NP_001037668.1	cytosolic juvenile hormone binding protein 36 kDa subunit
LOC101739069	264.3352	-2.9449	0.082776009	XP_021202615.2	inter-alpha-trypsin inhibitor heavy chain H4 isoform X1
LOC101743011	11.8302	-5.6420	0.082776009	XP_037872991.1	protein C1orf194 homolog isoform X2
LOC101746665	95.1712	-1.5307	0.082776009	XP_004928342.1	mitochondrial genome maintenance exonuclease 1 isoform X1
LOC119630461	12.3734	2.7814	0.082776009	XP_037875894.1	uncharacterized protein LOC119630461
LOC101735769	31.2552	3.9453	0.082776009	XP_021208533.1	uncharacterized protein LOC101735769 isoform X2
LOC101744832	9.5699	-6.2953	0.083378581	XP_004924666.1	uncharacterized protein LOC101744832 isoform X4

LOC101745657	68.5100	1.1045	0.083912695	XP_012543915.1	GPI mannosyltransferase 4
LOC101738839	17.1237	2.0951	0.085210763	XP_037871706.1	uncharacterized protein LOC101738839 isoform X1
LOC101739844	138.9796	-1.5134	0.085210763	XP_021207594.1	uncharacterized protein LOC101739844 isoform X2
LOC101736188	27.3774	-1.5633	0.085210763	XP_021205311.1	uncharacterized protein LOC101736188 isoform X1
LOC101739927	59.6730	0.9578	0.085210763	XP_021205518.1	calcium release-activated calcium channel protein 1 isoform X6
LOC110386390	64.2998	1.5187	0.08577822	NA	uncharacterized LOC110386390
LOC101738197	69.6863	1.1037	0.086230681	XP_004923056.1	X-linked retinitis pigmentosa GTPase regulator homolog isoform X1
LOC101745519	293.0657	-2.8152	0.086230681	XP_037872940.1	ommochrome-binding protein
LOC101741551	5.3601	-6.8174	0.08679326	XP_021208557.1	axoneme-associated protein mst101(2) isoform X2
LOC101737693	45.2579	-2.9303	0.087007922	XP_012548790.3	uncharacterized protein LOC101737693
LOC119630502	59.1089	1.6507	0.087222908	NA	uncharacterized LOC119630502
LOC733032	29.6923	1.3590	0.087273643	NP_001040489.1	DNA-3-methyladenine glycosylase I
LOC101745338	15.1679	-2.7498	0.087273643	XP_037875057.1	uncharacterized protein LOC101745338
LOC692805	225.5405	0.9370	0.087476451	NP_001040117.1	dynactin 4 protein
LOC101739109	48.2065	1.2760	0.088213949	XP_012548967.1	zinc finger protein 239-like
LOC101739651	59.7942	1.0446	0.089602135	XP_004930565.1	ras-related C3 botulinum toxin substrate 1
LOC101745551	18.9712	-5.1723	0.089998768	XP_037869336.1	NADPH oxidase 4 isoform X2
yellow-f	27.7045	-2.3569	0.090528766	XP_037868167.1	uncharacterized protein LOC101736658 isoform X1
LOC119630595	16.9372	-2.0769	0.090528766	XP_037876423.1	uncharacterized protein LOC119630595
LOC101742487	97.6843	1.1289	0.092696771	XP_021202497.2	G patch domain and ankyrin repeat-containing protein 1 homolog
LOC101743906	14.1428	2.0742	0.092921478	XP_037877760.1	uncharacterized protein LOC101743906
LOC101735747	41.8247	1.8792	0.093649561	XP_004927249.1	general transcription factor IIH subunit 3
LOC101745059	142.2931	-1.0092	0.094685088	XP_012544843.2	helicase SKI2W
LOC101736001	70.5506	1.0232	0.096350348	XP_037876759.1	vinculin
LOC101737161	171.7895	-0.9776	0.097239509	XP_037877751.1	cytoplasmic dynein 1 light intermediate chain 2
LOC110385005	36.7096	-1.1700	0.098164974	XP_037873331.1	uncharacterized protein LOC110385005 isoform X1
LOC101745477	375.8597	-0.9116	0.098164974	XP_012546787.2	protein mini spindles
LOC101737707	24.8642	1.8694	0.098164974	XP_004921931.1	chondroadherin
LOC101739178	28.7917	-1.5027	0.098164974	XP_037873042.1	leucine-rich repeat-containing protein 58 isoform X2
LOC732926	25.9132	-3.7294	0.098164974	NP_001040391.1	tetraspanin E118
LOC101743886	7.9724	-5.9175	0.098164974	XP_037873604.1	solute carrier family 22 member 5 isoform X2

LOC101740334	20.3930	-2.8520	0.098164974	XP_004930768.1	glucose-1-phosphatase
LOC732993	264.1469	0.7940	0.098164974	NP_001040454.1	ML-domain containing secreted protein precursor
LOC101735402	9.2815	-2.3197	0.098164974	XP_037877359.1	uncharacterized protein LOC101735402
LOC119630925	7.2879	-2.7615	0.098164974	XP_037877382.1	uncharacterized protein LOC119630925 isoform X1
LOC101735996	39.3110	-2.4281	0.098196633	XP_004924762.1	protein Spindly
Gata-beta	13.0925	-3.9695	0.098348774	XP_037871548.1	transcription factor BCFI isoform X3
LOC119629332	9.5577	2.4831	0.098615399	NA	U6 spliceosomal RNA
Nopp34	1086.4584	0.9385	0.098790808	NP_001037642.1	nucleolar phosphoprotein
LOC101743166	45.8563	-1.1822	0.099021105	XP_004924867.1	probable serine hydrolase
LOC101745112	12.1924	-2.5658	0.099021105	XP_037876635.1	telomere length regulation protein TEL2 homolog

Table S2. GO terms enriched among upregulated genes in the silk glands of selected *Bombyx mori* strains from CAR.

GO Term ID	GO description	*p*-value
	Biological Process	
GO:0006221	pyrimidine nucleotide biosynthetic process	0.013
GO:0006139	nucleobase-containing compound metabolic process	0.013
GO:0000266	mitochondrial fission	0.014
GO:0019509	L-methionine salvage from methylthioadenosine	0.014
GO:0006396	RNA processing	0.014
GO:0051726	regulation of cell cycle	0.016
GO:0006974	cellular response to DNA damage stimulus	0.016
GO:0006629	lipid metabolic process	0.016
GO:0016560	protein import into peroxisome matrix, docking	0.016
GO:0006355	regulation of transcription, DNA-templated	0.016
GO:0009058	biosynthetic process	0.017
GO:0005975	carbohydrate metabolic process	0.017
GO:0071704	organic substance metabolic process	0.017
GO:0016180	snRNA processing	0.017
GO:0007015	actin filament organization	0.017
GO:0033014	tetrapyrrole biosynthetic process	0.017
GO:0043039	tRNA aminoacylation	0.017
GO:0032367	intracellular cholesterol transport	0.028
GO:0055114	(obsolete) oxidation-reduction process	0.032
	Cellular Component	
GO:0000178	exosome (RNase complex)	0.014
GO:0005737	cytoplasm	0.014
GO:0005778	peroxisomal membrane	0.016
GO:0031209	SCAR complex	0.017
GO:0000439	transcription factor TFIIH core complex	0.017
GO:0034464	BBSome	0.032
	Molecular Function	
GO:0019205	nucleobase-containing compound kinase activity	0.013

GO:0009041	uridylate kinase activity	0.013
GO:0005515	protein binding	0.014
GO:0003723	RNA binding	0.014
GO:0046872	metal ion binding	0.014
GO:0016627	oxidoreductase activity, acting on the CH-CH group of donors	0.016
GO:0008531	riboflavin kinase activity	0.016
GO:0003824	catalytic activity	0.017
GO:0016868	intramolecular transferase activity, phosphotransferases	0.017
GO:0004812	aminoacyl-tRNA ligase activity	0.017
GO:0044877	protein-containing complex binding	0.017
GO:0030170	pyridoxal phosphate binding	0.017
GO:0080048	GDP-D-glucose phosphorylase activity	0.017
GO:0003870	5-aminolevulinate synthase activity	0.017
GO:0003676	nucleic acid binding	0.017
GO:0000166	nucleotide binding	0.017
GO:0004610	phosphoacetylglucosamine mutase activity	0.017
GO:0016747	acyltransferase activity, transferring groups other than amino-acyl groups	0.032
GO:0016491	oxidoreductase activity	0.033
GO:0008270	zinc ion binding	0.033
GO:0016757	glycosyltransferase activity	0.033
GO:0008641	ubiquitin-like modifier activating enzyme activity	0.033
GO:0033961	cis-stilbene-oxide hydrolase activity	0.049

Table S3. GO terms enriched among downregulated genes in the silk glands of selected *Bombyx mori* strains from CAR.

GO Term ID	GO description	*p*-value
	Biological Process	
GO:0005975	carbohydrate metabolic process	0.005
GO:0006351	transcription, DNA-templated	0.017
GO:0006526	arginine biosynthetic process	0.017
GO:0015031	protein transport	0.018
GO:0016052	carbohydrate catabolic process	0.018
GO:0006281	DNA repair	0.017
GO:0006003	fructose 2,6-bisphosphate metabolic process	0.020
GO:0009058	biosynthetic process	0.020
GO:0006000	fructose metabolic process	0.020
GO:0035434	copper ion transmembrane transport	0.021
GO:0032367	intracellular cholesterol transport	0.030
GO:0016192	vesicle-mediated transport	0.038
GO:0007186	G protein-coupled receptor signaling pathway	0.038
GO:0007166	cell surface receptor signaling pathway	0.038
GO:0055114	(obsolete) oxidation-reduction process	0.040
	Cellular Component	
GO:0005615	extracellular space	0.007
GO:0000439	transcription factor TFIIH core complex	0.017
GO:0016020	membrane	0.018
GO:0016021	integral component of membrane	0.021
GO:0030131	clathrin adaptor complex	0.038
	Molecular Function	
GO:0004553	hydrolase activity, hydrolyzing O-glycosyl compounds	0.005
GO:0008270	zinc ion binding	0.017
GO:0004833	tryptophan 2,3-dioxygenase activity	0.017
GO:0004055	argininosuccinate synthase activity	0.017
GO:0020037	heme binding	0.017
GO:0005524	ATP binding	0.017

GO:0046872	metal ion binding	0.017
GO:0050023	L-fuconate dehydratase activity	0.018
GO:0016787	hydrolase activity	0.018
GO:0016812	hydrolase activity, acting on carbon-nitrogen (but not peptide) bonds, in cyclic amides	0.018
GO:0016810	hydrolase activity, acting on carbon-nitrogen (but not peptide) bonds	0.018
GO:0003824	catalytic activity	0.019
GO:0003677	DNA binding	0.019
GO:0016788	hydrolase activity, acting on ester bonds	0.019
GO:0004518	nuclease activity	0.019
GO:0008890	glycine C-acetyltransferase activity	0.020
GO:0016740	transferase activity	0.020
GO:0003873	6-phosphofructo-2-kinase activity	0.020
GO:0030170	pyridoxal phosphate binding	0.020
GO:0019205	nucleobase-containing compound kinase activity	0.020
GO:0005375	copper ion transmembrane transporter activity	0.021
GO:0003796	lysozyme activity	0.027
GO:0004888	transmembrane signaling receptor activity	0.038
GO:0016491	oxidoreductase activity	0.040
